# Cnr2 Is Important for Ribbon Synapse Maturation and Function in Hair Cells and Photoreceptors

**DOI:** 10.3389/fnmol.2021.624265

**Published:** 2021-04-20

**Authors:** Luis Colón-Cruz, Roberto Rodriguez-Morales, Alexis Santana-Cruz, Juan Cantres-Velez, Aranza Torrado-Tapias, Sheng-Jia Lin, Guillermo Yudowski, Robert Kensler, Bruno Marie, Shawn M. Burgess, Olivier Renaud, Gaurav K. Varshney, Martine Behra

**Affiliations:** ^1^Department of Anatomy and Neurobiology, School of Medicine, University of Puerto Rico, San Juan, Puerto Rico; ^2^Genes & Human Disease Research Program, Oklahoma Medical Research Foundation, Oklahoma City, OK, United States; ^3^School of Medicine, Institute of Neurobiology, University of Puerto Rico, San Juan, Puerto Rico; ^4^Developmental Genomics Section, Translational and Functional Genomics Branch, National Human Genome Research Institute, National Institutes of Health, Bethesda, MD, United States; ^5^Cell and Tissue Imaging Facility (PICT-IBiSA, FranceBioImaging), Institut Curie, PSL Research University, U934/UMR3215, Paris, France

**Keywords:** ribbon synapse, cannabinoid receptor 2, hair cells, inner ear, retina, photoreceptors 3, endocannabinod system

## Abstract

The role of the cannabinoid receptor 2 (CNR2) is still poorly described in sensory epithelia. We found strong *cnr2* expression in hair cells (HCs) of the inner ear and the lateral line (LL), a superficial sensory structure in fish. Next, we demonstrated that sensory synapses in HCs were severely perturbed in larvae lacking cnr2. Appearance and distribution of presynaptic ribbons and calcium channels (Ca_v_1.3) were profoundly altered in mutant animals. Clustering of membrane-associated guanylate kinase (MAGUK) in post-synaptic densities (PSDs) was also heavily affected, suggesting a role for cnr2 for maintaining the sensory synapse. Furthermore, vesicular trafficking in HCs was strongly perturbed suggesting a retrograde action of the endocannabinoid system (ECs) via cnr2 that was modulating HC mechanotransduction. We found similar perturbations in retinal ribbon synapses. Finally, we showed that larval swimming behaviors after sound and light stimulations were significantly different in mutant animals. Thus, we propose that cnr2 is critical for the processing of sensory information in the developing larva.

## Introduction

The endocannabinoid system (ECs) is a well conserved modulator of almost all physiological systems, including the central and peripheral nervous systems (Joshi and Onaivi, 2019). Endocannabinoids mostly serve as retrograde messenger at various types of synapses throughout the brain, where they are synthesized in response to post-synaptic activation and bind pre-synaptic cannabinoid receptor 1 (CNR1) which in turn inhibits classical neurotransmitter release (Castillo et al., 2012). CNR1 expression and function was extensively described in the brain (Piomelli, 2003) but also in several peripheral organs (Maccarrone et al., 2015). By contrast, expression and function of the second well characterized cannabinoid receptor (CNR2) remains more elusive, in part due to interspecies differences at the genomic and tissue/organ expression levels (Liu et al., 2009). Recently, the previously accepted notion that CNR2 was mostly restricted to the immune system has been repeatedly challenged (Onaivi et al., 2012), and expression in the mammalian (Onaivi et al., 2006) and fish brain (Acevedo-Canabal et al., 2019) as well as associated behavioral changes were demonstrated. *Cnr2* expression was also described in adult sensory organs, specifically in the retina of several species (Bouchard et al., 2016) including fish (Cottone et al., 2013) in several cell types like photoreceptors, as well as in the rodent inner ear in several cell types like sensory hair cells (HCs) of the Organ of Corti (Martin-Saldana et al., 2016; Ghosh et al., 2018). Up-to-today, little is known about the developmental expression pattern or role of cnr2.

Hair cells are sensory mechanoreceptors which are found in the auditory part of the mammalian inner ear in the floor of the cochlear canal (=Organ of Corti), but also in sensory patches of the vestibular part, which is common to all vertebrates, and is comprised of 3 cristae and 2 maculae, that insure static and dynamic equilibrium (Bever and Fekete, 2002). In fish, in addition to a well conserved inner ear, there is an evolutionary linked superficial sensory organ named the lateral line (LL) that is composed of sensory patches called neuromasts (NMs) (Whitfield, 2002). NMs are stereotypically and symmetrically distributed on each side of the animal’s head (=anterior LL, aLL), trunk and tail (=posterior LL, pLL). In zebrafish, both the ear and the LL appear early in the embryo and mature rapidly, becoming functional in the 3 to 5-day post-fertilization (dpf) larva in a rostral to caudal gradient for the pLL (Ghysen and Dambly-Chaudiere, 2007). In mature HCs, the apical bundle of cilia are deflected by sound waves and head movements in the inner ear, and by water movements in the LL. The opening of mechanical ion channels triggers a graded membrane depolarization (Gillespie and Walker, 2001), that in turn activates the voltage- gated calcium channels in the latero-basal synapses, thus creating a calcium influx, that will drive the fusion of synaptic vesicles and release of neurotransmitter (glutamate) onto the innervating afferent fibers (Fuchs, 2005). HCs must transmit sound over a dynamic range of several orders of magnitude similar to how photoreceptors transmit light signals, meaning that changes of intensity in the stimulus are encoded by adjusting the tonic (sustained) rate of neurotransmitter release (Tom Dieck and Brandstatter, 2006). A phasic (transient) rate of vesicle release was also demonstrated in retinal cones exposed to light when detecting variation in light intensity (Rabl et al., 2005; Jackman et al., 2009). Graded synaptic output requires the release of up to several thousand synaptic vesicles/second. This is made possible by signature structures that are exclusively found in sensory receptor cell synapses that are called ribbons or dense bodies.

Ribbons are anchored to the basolateral membrane directly adjacent to synaptic cluster of L-type voltage gated calcium channels and are surrounded by tethered glutamate-filled vesicles (Moser et al., 2006; Matthews and Fuchs, 2010). The ribbon itself is a dynamic electron dense structure that can adopt different shapes, typically forming plates in retinal cells (Tom Dieck and Brandstatter, 2006), and spheroids in HCs (Sterling and Matthews, 2005; Moser et al., 2006). The main constituent of ribbons is the RIBEYE protein that is encoded by the *CTBP2* gene with a unique N-terminal A domain that does not share homology with any other known protein, and a C-terminal B domain identical to the CTBP2 protein (Nicolson, 2015). Furthermore, both the size and number of ribbons per sensory cell vary depending on the species (Nouvian et al., 2006), the cell type (Nouvian et al., 2006), the position in the sensory epithelium (Johnson et al., 2008), the developmental stage (Safieddine et al., 2012; Wong et al., 2014), and the sensory activity (Liberman et al., 2011). In the LL of zebrafish larva, mature HCs arise as early as 3 dpf, in which three to four spherical ribbons (diameter ∼200 nm) neatly arranged in the basolateral portion can be visualized (Nicolson, 2015; Lv et al., 2016; Suli et al., 2016; Sheets et al., 2017). Tethered spherical (diameter ∼20 nm) glutamate-filled vesicles surround the ribbon body. A subset in direct contact with the cytoplasmic membrane is docked which represents the ready-to-release pool (RRP) (Moser et al., 2006; Nouvian et al., 2006; Schmitz, 2009). Originally thought to work as a conveyor belt, the ribbon is now viewed as a safety belt slowing down and organizing compound fusion of vesicles at the synapses (Parsons and Sterling, 2003; Matthews and Fuchs, 2010).

A number of additional proteins have been described at the ribbon synapse, some of them are common to classical synapses, and others exclusive (for review Safieddine et al., 2012; Moser et al., 2020) like the highly conserved L-type calcium channels (Ca_v_1.4 in the retina and Ca_v_1.3 in HCs). Absence of Ca_v_1.3 causes profound deafness in humans (Baig et al., 2011), mice (Brandt et al., 2005), and zebrafish (Sidi et al., 2004). Furthermore, a close relationship between Ca_v_1.3 distribution and ribbon size and position was carefully characterized (Sheets et al., 2011, 2012, 2017) confirming the crucial role of both for proper synaptic exo- and endocytosis (Frank et al., 2010; Sheets et al., 2011). A classical approach to study synaptic vesicular trafficking is the application of FM 1-43, a styryl probe that fluoresces brightly when taken up in a membrane, that was widely used in a variety of preparations like neuro-muscular junctions (Betz et al., 1992; Henkel et al., 1996; Kuromi and Kidokoro, 1998; Reid et al., 1999), cultured hippocampal neurons (Ryan and Smith, 1995), and retinal bipolar cells (Neves and Lagnado, 1999). However, in HCs, FM 1-43 rapidly penetrates the apical ciliated region via two proposed mechanisms: apical cuticular endocytosis (Seiler and Nicolson, 1999; Self et al., 1999; Meyer et al., 2001; Griesinger et al., 2002, 2004), and passive diffusing through mechanotransduction channels (MET) (Nishikawa and Sasaki, 1996; Gale et al., 2000; Gale et al., 2001; Meyers et al., 2003) and ion channels (Crumling et al., 2009). Thus, FM 1-43 will also report constitutive vesicle trafficking (Kachar et al., 1997) which can potentially mask synaptic trafficking (Saito, 1983; Siegel and Brownell, 1986). Nevertheless, FM 1-43 was extensively used for assessing mechano-transduction during development (Seiler and Nicolson, 1999; Geleoc and Holt, 2003; Seiler et al., 2004; Sidi et al., 2004), intracellular apico-basal trafficking (Kaneko et al., 2006; Harasztosi and Gummer, 2019) and synaptic recycling in HCs (Kamin et al., 2014).

We found that the *cnr2* gene was expressed in all HCs of the inner ear and the LL, starting at 3 day post-fertilization (dpf) onward and hypothesized that it had an important role in neurotransmission in those sensory mechanoreceptors. In *cnr2* loss-of-function animals (*cnr2^*upr*1^*) that we previously generated (Acevedo-Canabal et al., 2019), HCs in NMs of the LL were not strongly affected in their development, regeneration, or innervation. However, we found profound perturbation in the size and distribution of synaptic ribbons which was more evident in mature HCs. Furthermore, we showed alteration in the distribution of Ca_v_1.3 channels, and we demonstrated that the alignment of pre- and post-synaptic elements in afferent dendrites was compromised. Using TEM and live FM 1-43 imaging, we showed that the trafficking of neurotransmitter vesicles was affected, suggesting a putative cnr2 mediated retrograde activity of the ECs in HCs. We further observed perturbation of the ribbon synapses in the retina of *cnr2^*upr1/upr*1^* animals, raising the possibility of a mechanism that is common to all sensory synapses. Finally, we established that mutant larvae were more sensitive to sound stimulation when also exposed to light, and conversely more sensitive to light when previously exposed to sound, thus providing a link between sensory tasks in a cnr2-deficient context and illustrating the physiological relevance of our findings.

## Results

### Cnr2 Is Strongly Expressed in Hair Cells (HCs) of the LL and the Inner Ear

**FIGURE 1:**
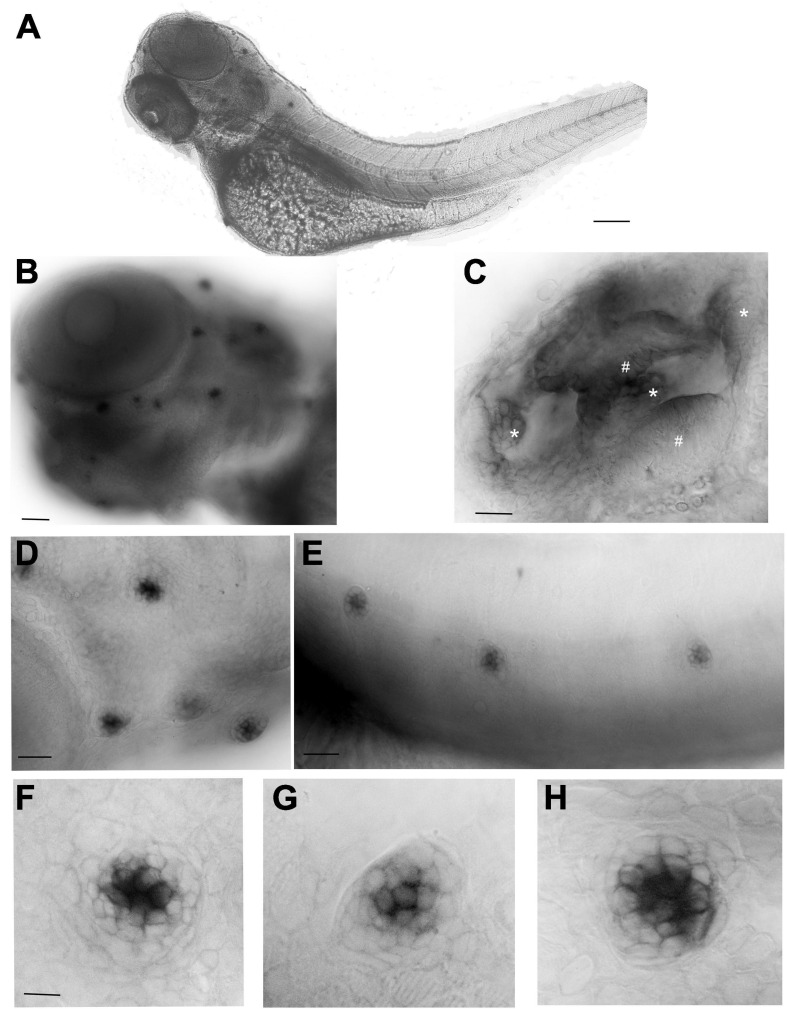
Whole mount *in situ* hybridization (WISH) with an antisense probe against *cnr2* in 3-dpf wildtype larvae. **(A)** Lateral view of a whole larva. **(B)** Magnification of the head region showing head neuromasts (NMs) and inner ear expression. **(C)** Inner ear showing cnr2 expression in all three cristae (*) and two maculae (^#^). **(D)** Magnification of cranial NMs in the anterior lateral line (aLL) **(E)**. Magnification of trunk NMs in the posterior LL (pLL). **(F–H)** Individuals NMs show *cnr2* expression that is restricted to the centrally located HCs. Scale bars: in **(A)** = 1500 microns; **(B)** = 300 microns; **(C)** = 50 microns; **(D)** = 20 microns; **(E)** = 150 microns; in panels **(F–H)** = 25 microns.

To define the *cnr2* expression pattern during development, we performed whole mount *in situ* hybridization (WISH) with an antisense probe against *cnr2* in 3 and 5-day post-fertilization (dpf, *n* = 30/stage) wildtype animals. At 3-(Figure 1) and 5-dpf (Supplementary Figure 1), we found a strong hair cell (HC)-specific expression in all neuromasts (NMs) of the lateral line (LL, Figures 1A–H), as well as in all sensory patches of the inner ear, namely maculae (Figure 1C, #) and cristae (Figure 1C, ^∗^).

To elucidate the putative role of cnr2 in those sensory epithelia, we used the stable loss-of-function mutant line (*cnr2^*upr*1^*) that we had previously generated (Acevedo-Canabal et al., 2019), to monitor HC development and regeneration in the LL in the absence of cnr2 (Supplementary Figure 2). We visualized functional HCs using YOPRO-1, a live dye that gets rapidly incorporated into HCs. We counted HCs in successive developmental stages (Supplementary Figures 2A,B), namely at 2-, 3-, 4-, 5-, 6-, and 7-dpf (with a minimum of *n* = 22/genotype/stage) wildtype and *cnr2^*upr*1/upr1^* larvae. The overall development of the LL and the gross morphology of NMs seemed unaffected, but the average HCs’ number/NM was slightly but significantly reduced in mutant larvae starting at 3-dpf and onward. Next, we triggered synchronized HC-destruction using copper treatments in 5-dpf wildtype and mutant larvae and counted regenerating HCs at 0-, 24-, 48-, and 72-h post-treatment (hpt, Supplementary Figure 2C, *n* = 23/genotype/hpt). We found that HC regeneration was not significantly affected until 48-hpt and only slightly decreased thereafter. Taken together, *cnr2* was strongly expressed in HCs of the LL but when absent, both HC development and regeneration were only moderately affected, suggesting cnr2 was not indispensable for either.

### Cnr2 Expression Affects Ribbon Synapses in Mature HCs of the LL and the Inner Ear

To address the putative role of cnr2 in HCs, we focused on the LL and assessed efferent and afferent innervation of NMs. We immunolabelled 5-dpf wildtype and *cnr2^*upr*1/upr1^* larvae with two classical antibodies (Abs): Znp1, to stain motor neurons and terminals, and Myosin7 (Myo7) to label HCs (Supplementary Figure 3A, *n* = 15/genotype). We found no overt differences in the efferent innervation of NMs. In parallel, we immunolabelled sensory neurons and ribbons synapses in HCs using Zn12 and Ribeye b Abs (Supplementary Figure 3B, *n* = 12/genotype). The overall sensory innervation appears intact (Zn12 in magenta). However, the Ribeye b staining (green) was strongly perturbed in mutant NMs, suggesting that ribbon synapses were defective in the absence of cnr2 expression (compare Supplementary Movie 1: Zn12-Rib-Wt with Supplementary Movie 2: Zn12-Rib-cnr2).

**FIGURE 2:**
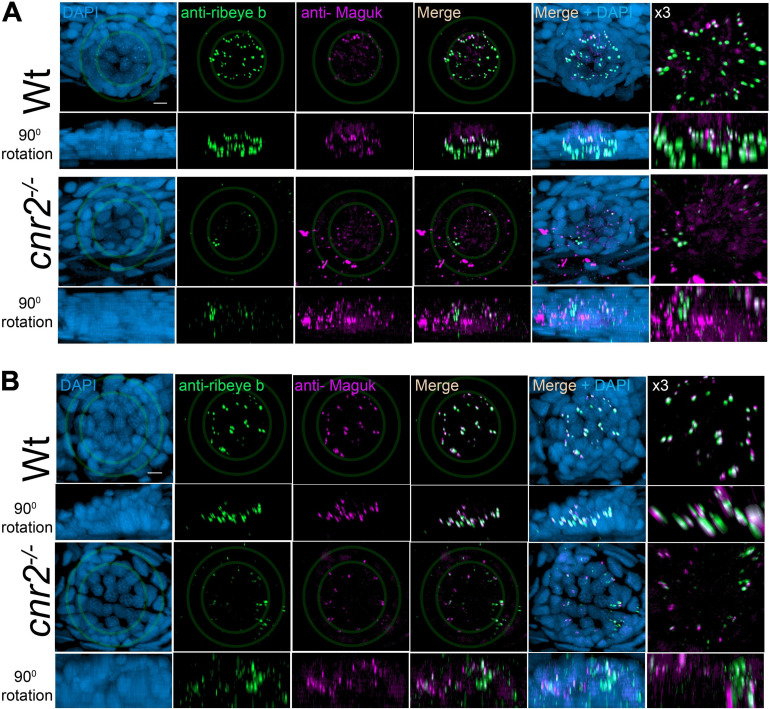
Immunofluorescent labeling of ribbon synapses (Ribeye b) in HCs and post-synaptic densities (PSDs, Maguk) in afferent dendrites in NMs of wildtype and *cnr2*^*upr1/upr1*^ 5-dpf larvae. **(A)** Cranial NMs (IO2) from the aLL, in top views (first and third rows) and lateral views in 90^*o*^ image rotations (second and fourth rows). Corresponding central regions were magnified x3 (right panels). Larvae were stained with an anti-Ribeye b AB (green) which is labeling presynaptic ribbon synapses, and an anti-Maguk AB (Magenta) which is labeling post-synaptic densities, and counterstained with DAPI **(B)**. Same immuno-labeling and imaging in trunk NMs (L3) from the pLL. Scale bar = 20 microns. Limits of MNs and inner HCs are outlined (gray outer and inner circles, respectively).

To further analyze the sensory synapses, we co-immunolabelled 5-dpf wildtype (*n* = 20) and mutant larvae (*n* = 23) with Abs against Ribeye b, and against the scaffolding protein membrane-associated guanylate kinase (Maguk) which forms foci in post-synaptic densities (PSDs) of sensory neurites (Sheets et al., 2011; Lv et al., 2016; Figure 2). In wildtype NMs (first and second rows) in both the aLL (Figure 2A) and the pLL (Figure 2B), we found that Ribeye b puncta (green) appeared neatly organized and localized to the laterobasal portion of HCs (more visible in 90^0^ rotation of the acquired images, second rows in Figures 2A,B). On average, we found 3 to 4 evenly shaped and sized puncta/HC as previously described (Sheets et al., 2014, 2017). By contrast, in NMs of cnr2 homozygote mutants (third and fourth rows in Figures 2A,B), we found much less Ribeye b puncta, which were highly variable in shape and size, unorganized and not always localized to the basal portion of HCs. Furthermore, in the PSDs of wildtype NMs and as previously described, we found focalized Maguk staining (magenta in first and second rows in Figures 2A,B) which was mostly located in close vicinity to the Ribeye b puncta, thus forming bi-labeled clusters (most visible in the X3 magnification, right column). By contrast, in mutant NMs, Maguk staining was often weak and diffuse with few foci which when present were rarely in the close vicinity of Ribeye b puncta. Taken together, this was strongly suggesting that the sensory synapses were perturbed both in the presynaptic zone in HCs and in the post-synaptic dendrites, and the alignment of synaptic elements was defective.

**FIGURE 3:**
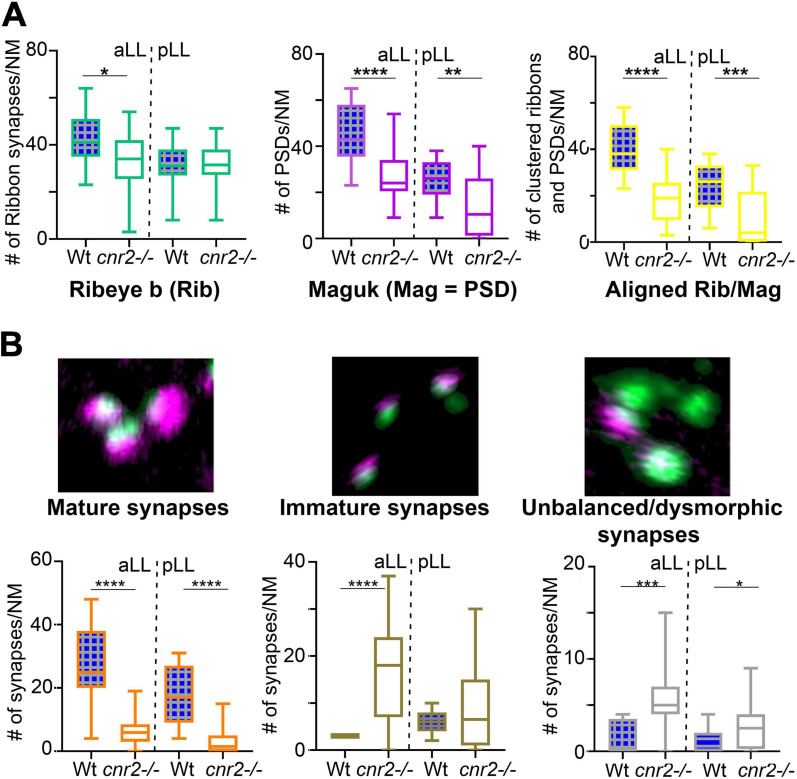
Quantification of individual Ribeye b and Maguk puncta and aligned/clustered Ribeye b and Maguk puncta. **(A)** Quantification of Ribeye b puncta (Rib, left panel), Maguk puncta (Mag, central panel) and the two clustered/aligned (Rib/Mag, right panel) in NMs of the aLL and pLL. **(B)** Visual assessment of the maturity of synapses based on the size, shape and relative proportion of Rib and Mag clusters. Example of mature (left panel), immature (central panel) or unbalanced/dysmorphic (right panel) synapses are shown. Scale bar in panel **(B)** (for all 3 images) = 1 micron. Whisker boxes show all values with minimum and maximum. Significance is represented as * (*p < 0.05; **p < 0.001; ***p < 0.003; and ****p < 0.0001).

Next, we quantified Ribeye b puncta, Maguk foci and the overlapping of both in wildtype (*n* = 4) and *cnr2^*upr*1/upr1^* larvae (*n* = 7) in NMs of the aLL (NM_WT_ *n* = 17; NM_cnr__2_ *n* = 17) and pLL (NM_WT_ *n* = 19; NM_cnr__2_ *n* = 20). For Ribeye b, we counted all puncta regardless of size (Figure 3A, left graph) and found that the number of ribbon synapses (RS) was significantly decreased in mutant NMs in the aLL (RS/NM_WT_ = 43.35 vs. RS/NM_cnr__2_ = 33.94, *p* = 0.0256), but not in the pLL (RS/NM_WT_ = 30.99 vs. RS/NM_cnr__2_ = 31.30, *p* = 0.8964), possibly because trunk and tail NMs were less mature than cranial NMs. For Maguk, we counted all foci regardless of the size (middle graph) and found that the number of PSDs was significantly reduced in all NMs of aLL (PSD_WT_/NM = 45.47 vs. PSD_cnr__2_/NM = 26.59, *p* < 0.0001), and pLL (PSD_WT_/NM = 25.74 vs. PSD_cnr__2_/NM = 14.00, *p* = 0.0037). Finally, we scored all overlapping staining of Ribeye b and Maguk regardless of the respective size of either (right graph) and we found that the number of RS clustered with PSDs was greatly reduced in aLL (RS/PSD_WT_/NM = 40.24 vs. RS/PSD_cnr__2_/NM = 18.82, *p* < 0.0001) and in pLL (RS/PSD_WT_/NM = 23.95 vs. RS/PSD_cnr__2_/NM = 10.40, *p* < 0.0003) of *cnr2^*upr*1/upr1^*animals. Furthermore, only in wildtype NMs did we find the previously described mature aligned synapses which are forming evenly sized, balanced clusters (Lv et al., 2016; Sheets et al., 2017). In the mutant NMs, when we did find bi-labeled clusters they appeared mostly uneven in size and labeling, presumably reflecting mostly immature synapses (Figure 2, right column). Thus, we next subdivided the co-labeled clusters into three categories: (1) mature (Figure 3B, left graph), (2) immature when either staining in the cluster was strongly unbalanced (middle graph), (3) and unbalanced with either staining strongly dysmorphic (right graph). Mature synapses (S) were clearly less abundant in mutant NMs of the aLL (S_WT_/NM = 26.65 vs. S_cnr__2_/NM = 6.50, *p* < 0.0001) and pLL (S_WT_/NM = 17.95 vs. S_cnr__2_/NM = 3.80, *p* < 0.0001). Immature synapses (IS) were rarely found in wildtype NMs from the aLL, but their number was strongly increased in mutant animals (IS_WT_/NM = 3.00 vs. IS_cnr__2_/NM = 16.24, *p* < 0.0001). Notably, in the pLL, IS numbers were not significantly different in wildtype vs. mutant NMs (IS_WT_/NM = 6.316 vs. IS_cnr__2_/NM = 8.800, *p* < 0.2472), possibly reflecting the ongoing antero-posterior maturation of NMs in the LL. Finally, we only found abnormal synapses (AS) in mutant NMs of the aLL (AS_WT_/NM = 1.41 vs. AS_cnr__2_/NM = 5.41, *p* = 0.0005) and pLL (AS_WT_/NM = 1.16 vs. AS_cnr__2_/NM = 2.90, *p* = 0.0192). Taken together, in the absence of cnr2 expression all HCs of the LL presented strongly altered Ribeye b staining as well as perturbed Maguk staining in the PSDs in afferent neurites. Alterations were generally stronger in the more mature NMs of the aLL, suggesting that cnr2 is possibly involved in the maturation of ribbon synapses in HCs.

**FIGURE 4:**
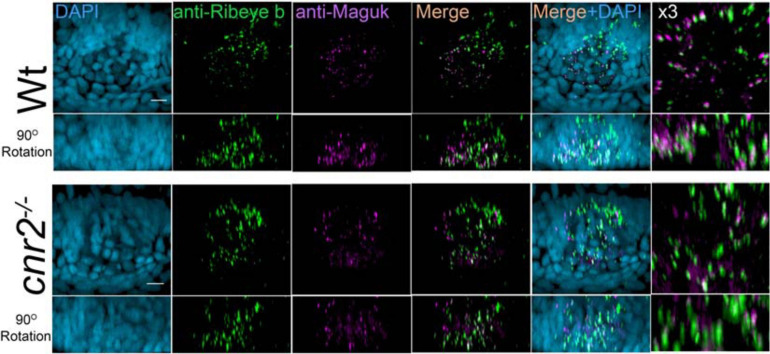
Immunofluorescent labeling of ribbon synapses (Ribeye b) in HCs and PSDs (Maguk) in afferent dendrites of sensory epithelia of the inner ear in wildtype and *cnr2*^*upr1/upr1*^ 5-dpf larvae. Top views (first and third rows) and lateral views in 90^*o*^ image rotations (second and fourth rows) with corresponding central regions magnified x3 (right panels) of macula of wildtype (two top rows) and mutant larvae (two bottom rows) stained with an anti-Ribeye b AB (green) which is labeling presynaptic ribbon synapses, and an anti-Maguk AB (Magenta) which is labeling post-synaptic densities, and counterstained with DAPI. Scale bars = 20 microns.

To verify if HCs of sensory epithelia in the inner ear were also affected, we imaged and analyzed Ribeye b and Maguk staining in maculae (Figure 4) and cristae (not shown) of wildtype (two top rows) and larvae lacking cnr2 (two bottom rows). Maculae at this stage, followed closely the curved shape of the inner ear with most mature synapses located on the ventral portion of the epithelium, as evidenced by more Ribeye b and Maguk overlapping clusters (see Supplementary Movie 3: Rib-Maguk macula-Wt). This apparent maturation gradient was also present in mutant maculae, but overall, both Ribeye b and Maguk staining appeared irregular. Notably, Ribeye b puncta (green) Maguk foci (magenta) were more uneven in size, and the overlapping clusters were strongly reduced in number and size in mutant larvae (right column and Supplementary Movie 4: Rib-Maguk macula-cnr2). Therefore, cnr2 seemed also required for proper maturation of ribbon synapses in HCs of the inner ear, suggesting a regulatory mechanism common to all HCs in zebrafish larva.

### Cnr2 Controls Presynaptic Calcium Channels (Ca_v_1.3) Distribution in All HCs

**FIGURE 5:**
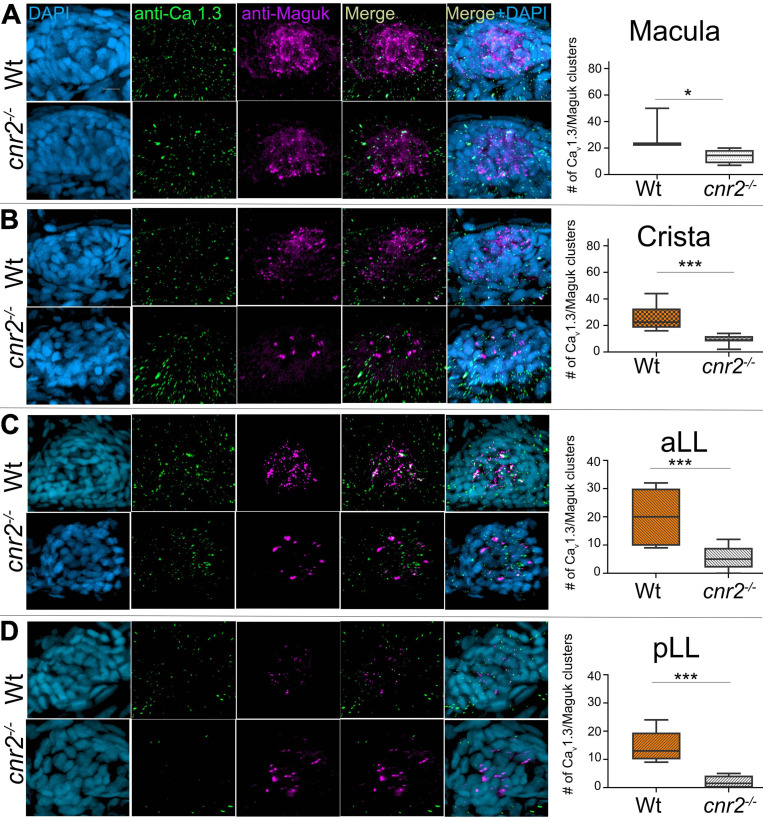
Immunofluorescent labeling of pre-synaptic calcium channels (Ca_v_1.3) and post-synaptic densities (PSDs, Maguk) and quantification of clustering in HCs of 5dpf wildtype and cnr2*^*upr1/upr1*^* larvae. **(A)** Top views of wildtype (top lane) and mutant (lower lane) macula that were stained with anti-Ca_v_1.3 (green) and anti-Maguk (magenta) and counterstained with DAPI. Clusters of Cav1.3 puncta and Maguk foci were quantified. **(B)** Top views of wildtype and mutant cristae. **(C)** Top views of wildtype and mutant NMs from the aLL (IO2). **(D)** Top views of wildtype and mutant NM form the pLL (L1). Scale bar = 20 microns. Whisker boxes show all values with minimum and maximum. Significance is represented as * (*p < 0.05 and ***p < 0.003).

To explore if Ca_v_1.3 distribution was affected in HCs of mutant larvae, we co-immunolabelled 5dpf wildtype (*n* = 20) and mutant larvae (*n* = 23) with Abs against Ca_v_1.3, and Maguk. We imaged all sensory epithelia of the inner ear as well as NMs of the aLL and pLL (Figure 5). We found that Maguk staining (Magenta) was more diffuse and generally less focalized in all mutant sensory epithelia. Strikingly, Ca_v_1.3 staining (green) appeared also strongly diminished in all mutant structures. Next, we quantified the number of aligned Ca_v_1.3 clusters and Maguk foci (graphs in right column). In all epithelia, we found strikingly less alignment between pre- and post-synaptic elements. In wildtype maculae, we found an average of 32 aligned clusters vs. 13.83 in mutants (graph in Figure 5A, *p* = 0.0298). In wildtype cristae, we found an average of 25.5 clusters vs. 9 in mutants (graph in Figure 5B, *p* = 0.001). In wildtype NMs from the aLL, we found an average of 20 clusters vs. 5.55 in mutants (graph in Figure 5C, *p* = 0.0009). And finally, in wildtype NMs of the pLL we found an average of 14.4 clusters vs. 2 in mutants (graph in Figure 5D, *p* = 0.0009). Thus, the Ca_v_1.3 distribution was profoundly altered in all sensory epithelia, suggesting an important role for cnr2 for proper presynaptic localization of Ca_v_1.3.

**FIGURE 6:**
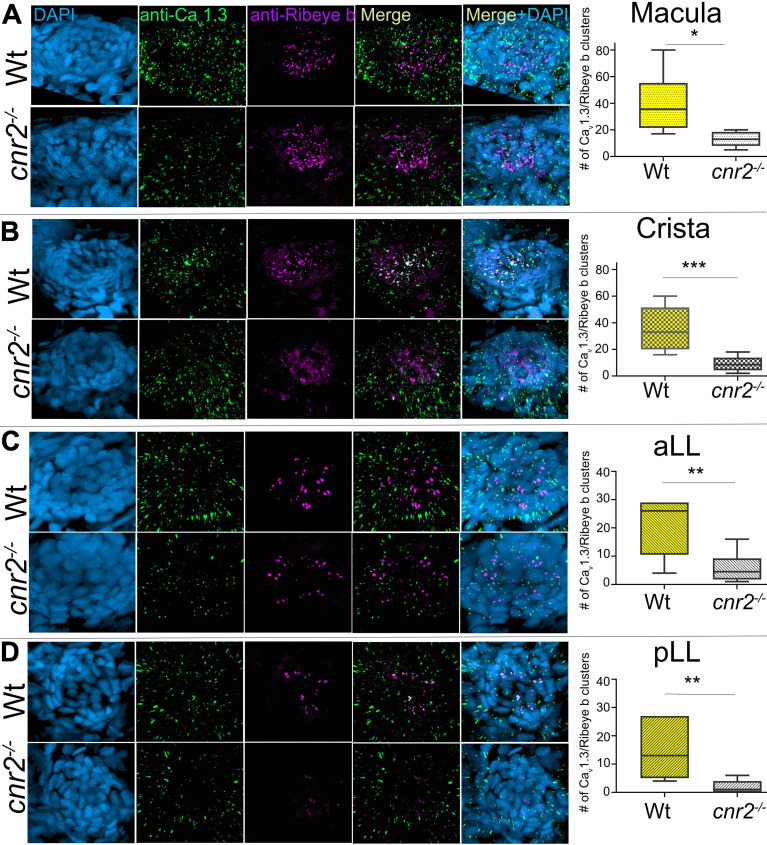
Immunofluorescent labeling of pre-synaptic calcium channels (Ca_v_1.3) and ribbon synapses (Ribeye b) and quantification of clustering in HCs of 5dpf wildtype and cnr2*^*upr1/upr1*^* larvae. **(A)** Top views of wildtype (top lane) and mutant (lower lane) macula that were stained with anti-Ca_v_1.3 (green) and anti-Ribeye b (magenta) and counterstained with DAPI. Clustered Cav1.3 and Ribeye b were quantified in maculae. **(B)** Top views of wildtype and mutant cristae and corresponding quantification of Cav/Rib clusters in cristae. **(C)** Top views of wildtype and mutant NMs from the aLL (IO2) and corresponding quantification of Cav/Rib clusters in NMs of the aLL. **(D)** Top views of wildtype and mutant NMs from the pLL (L1) and corresponding quantification of Cav/Rib clusters in NMs of the pLL. Scale bar = 20 microns. Whisker boxes show all values with minimum and maximum. Significance is represented as * (*p < 0.05; **p < 0.001; and ***p < 0.003).

Next, we examined the expression of Ca_v_1.3 in respect to synaptic ribbons (Figure 6). As previously described, Cav1.3 staining (green) was strongly reduced, and ribbons synapses appeared deeply perturbed (magenta). We quantified colocalization of both in all epithelia. In wildtype maculae, we found the greatest variation in wildtype with an average of 39.67 colocalized clusters. However, we found significantly less in mutant maculae (graph in Figure 6A, 13 clusters, *p* = 0.0183). In wildtype cristae, we found an average of 35.33 clusters vs. 9.37 in mutants (graph in Figure 6B, *p* = 0.0007). In wildtype NMs from the aLL, we found an average of 20.33 clusters vs. 5.83 in mutants (graph in Figure 6C, *p* = 0.0061). And finally, in wildtype NMs of the pLL we found an average of 15 clusters vs. 1.857 in mutants (graph in Figure 6D, *p* = 0.0039). Thus, confirming that both ribbon synapses as well as Ca_v_1.3 distribution were profoundly altered in all sensory epithelia, underlying the importance of cnr2 for proper maturation of sensory synapses.

### Cnr2 Modulates Distribution of Neurotransmitter (Glutamate) Vesicles in HCs

**FIGURE 7:**
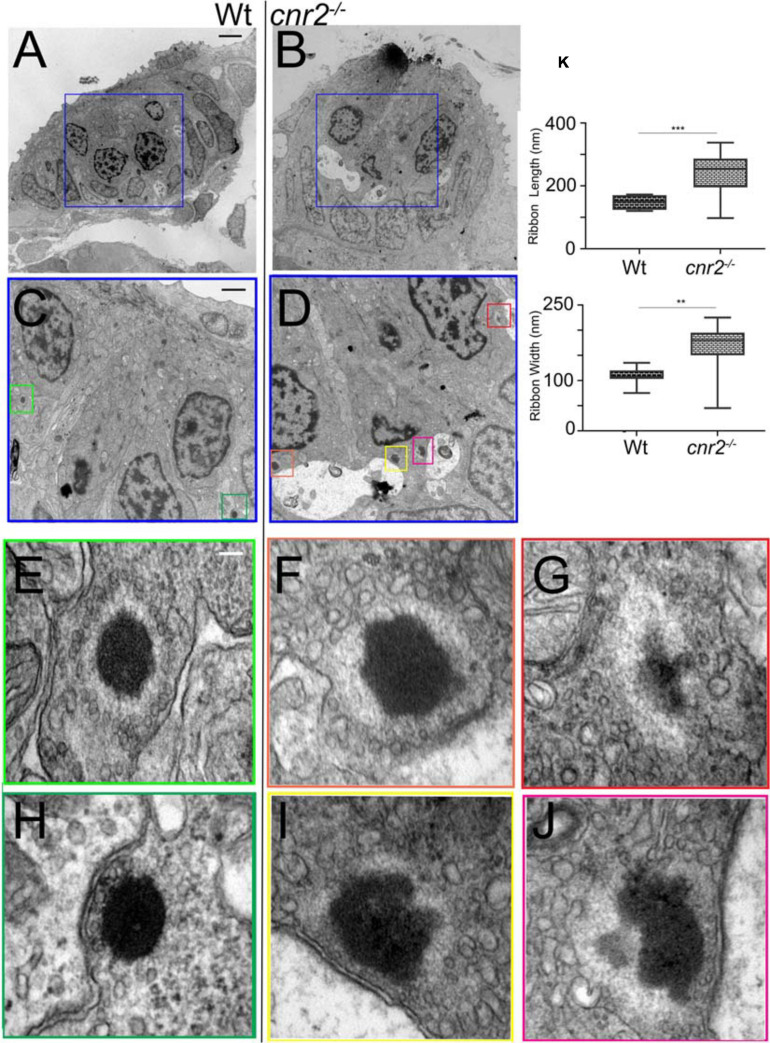
Transmission Electron microscopy (TEM) images in NMs of 5dpf wildtype and *cnr2*^*upr1/upr1*^mutant larvae. **(A,B)** Lower magnification showing the entire section through a wildtype (left) and a *cnr2*^*upr1/upr1*^ NM (right) with central ciliated HCs surrounded by support cells and mantle cells. HCs afferent innervation is visible in both sections (light stained structures, red*). **(C,D)** Higher magnifications of the corresponding areas (blue squares) in successive sections of the wildtype and the cnr2 NMs respectively, focusing on the synaptic regions displaying ribbon synapses. **(E–H)** Higher magnifications of each corresponding area (green squares) highlighting two wildtype ribbon synapses with tethered vesicles at equidistance that appear highly organized and evenly shaped (white arrowheads). Docked vesicles (white arrows) are also visible (in panel **H**) in close vicinity to the PSD (blue arrow). **(F,G,I,J)** Higher magnifications of each corresponding area (orange, red, yellow, and burgundy squares) highlighting 4 different aberrant ribbon synapses in a *cnr2*^*upr1/upr1*^ NM. The ribbons appear bigger and misshapen. The surrounding vesicles are at various distances from the central body, and of different size and shape (magenta arrowheads). Some docked vesicles (magenta arrows in panels **F,I,J**) are visible and so is the PSD (blue arrows in panels **(F,I,J)**. **(K)** Quantification of the ribbons’ length (top graph) and width (lower graph). Whisker boxes show all values with minimum and maximum. Significance is represented as * (**p < 0.001 and ***p < 0.003). Scale bars: in panels **(A,B)** = 5 microns; **(C,D)** = 1 micron; **(E–J)** = 50nm.

To examine the ultra-structure of ribbon synapses and to verify if the distribution and morphology of neurotransmitter vesicles were also affected, we analyzed NMs of 5dpf wildtype (*n* = 3) and cnr2 homozygote (*n* = 3) larvae using TEM (Figure 7). In all HCs of wildtype (Figures 7A,C,E,H) and mutant (Figures 7B,D,F,G,I,J) NMs, we located ribbon synapses in the basolateral walls of the HCs and in close vicinity to innervating dendrites (red stars). At higher magnification and as expected, in wildtype HCs we repeatedly found ribbon synapses which were regular in shape and size (two representative examples are shown in Figures 7E,H). Likewise, the ribbon bodies were surrounded by regularly shaped and sized (∼20 nm) spherical vesicles (Figure 7E, white arrowheads). All were tethered at a constant distance (∼10 nm) and docked vesicles were also distinguishable (Figure 7H, white arrows). By contrast, in the mutant HCs, all the ribbon synapses that we found were presenting electron dense cores that were bigger and misshapen (representative illustrations are shown in Figures 7F,G,I,J). We quantified the length and the width of the dense bodies (Figure 7K) and found that both were significantly different. The ribbons in wildtype HCs were on average 148 nm long and 108 nm wide, vs. 239.5 nm (*p* = 0.0006) and 166.1 nm (*p* = 0.0016) respectively. Strikingly, the surrounding vesicles were often not spherical, highly variable in size and shape (magenta arrowheads) and positioned at variable distance from the dense ribbon core. However, in most mutant HCs, the PSD was still clearly visible (blue arrows) and so were docked vesicles (magenta arrows in Figures 7F,I,J), suggesting at least partial synaptic function. Taken together, the ultra-structure of the ribbon synapses and the surrounding vesicles was profoundly perturbed in animals lacking cnr2, pointing to an important role in the ultra-structural organization at the sensory synapse.

Next, we assessed the integrity of ribbons synapses in the retina of wildtype and cnr2 loss of function larvae. We focused on the outer plexiform layer (OPL) where ribbon synapses in cones pedicles (Supplementary Figures 4A,D) and rod spherules (not shown) are abundant (Linberg and Fisher, 1988; Sterling and Matthews, 2005). The base of the ribbon synapse is anchored at the presynaptic active zone via the arciform density, a retina specific structure where a number of synaptic proteins like Bassoon, RIM2, ubMunc13-2, ERC2/CAST1 are located (for review Lagnado and Schmitz, 2015). These proteins are important for the proper localization of L-type calcium channels (Ca_v_1.4) at the presynaptic plasma membrane. In wildtype retina, we repeatedly found ribbons synapses with clearly defined arciform densities (Supplementary Figures 4A,D, blue arrows), opposing equally well-defined electron dense presynaptic plasma membrane (blue arrowheads). The vesicles in the vicinity of the ribbons appeared abundant and evenly sized (white arrowheads in Supplementary Figures 4A,D). However, in mutant retina Supplementary Figures 4B,C,E,F), most ribbons had incomplete or poorly defined arciform densities (magenta arrows), and the presynaptic plasma membrane appeared blurry and weakly delineated (magenta arrowheads), suggesting disturbed presynaptic active zones. Furthermore, neurotransmitter vesicles in the vicinity of the ribbon synapses appeared scattered, more interspersed, and of variable size (white arrowheads in Supplementary Figures 4B,C,E,F) suggesting that vesicular trafficking was also perturbed. Taken together, the absence of functional cnr2 seemed to affect the ultra-structure of retinal sensory synapses pointing to a putative regulatory role in the eye, reminiscent of our findings in the inner ear and LL.

### Cnr2 Affects Vesicular Trafficking in HCs

**FIGURE 8:**
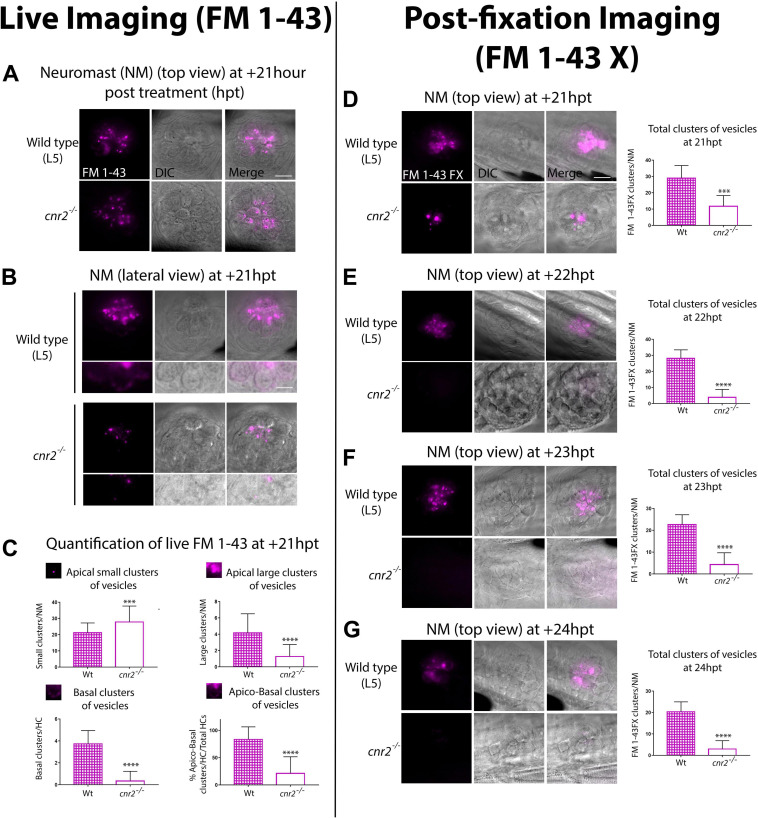
Live **(A–C)** and post-fixation **(D–G)** imaging in wildtype and mutant NMs after FM 1-43X treatment to visualize endocytic vesicular trafficking. **(A)** Top views and **(B)** lateral views of NMs of the pLL (L5) at +21 hpt in wildtype (top row) and *cnr2*^*upr1/upr1*^(bottom row). Respective inserts below each panel show the magnified basal compartments of the HCs. **(C)** Quantification of FM-43 residual staining at +21 hpt in wildtype (checkered bars) and *cnr2*^*upr1/upr1*^ (open bars) of apical small clusters (top left graph), apical large clusters (top right graph), basal clusters (bottom left graph) of vesicles, and% of apical and basal stained HCs/total HCs (bottom right graph). **(D–G)**. Top views of NMs of the pLL in wildtype (top panels) and *cnr2*^*upr1/upr1*^ (bottom panels), showing the vesicular distribution of FM 1-43X with the respective quantification o number of clusters of vesicles/NM (right graphs), at +21 hpt **(D)**, +22 hpt **(E)**, +23 hpt **(F)**, and +24 hpt **(G)**. Scale bars: = 20 microns in panels **(A,D)**, and = 10 microns in panel **(B)**. Significance is represented ***p < 0.003 and ****p < 0.0001.

To assess the integrity of vesicular trafficking in HCs, we briefly exposed 4 dpf wildtype and mutant larvae to FM 1-43 live dye and subsequently imaged NMs *in vivo* (L5 in the pLL) from wildtype and mutant animals (*n* = 3/genotype) during 24 h post-treatment (hpt) (Figures 8A-C). At 1-hpt, the staining did not appear different in wildtype and mutant HCs of the NMs (Supplementary Figure 5, at 1 hpt) suggesting that mechanotransduction was intact in mutant HCs. This result was in line with *cnr2^*upr*1/upr1^* larvae showing no overt behavioral phenotype after sound and vibration stimulation (LC and MB unpublished data).

However, over time (Supplementary Figure 6) and most visible from 18 hpt onward, residual FM 1-43 staining was weaker in mutant HCs (Figure 8A, top view at +21 hpt). To narrow down the cellular localization of vesicles, we imaged HCs in lateral views (Figure 8B). In wildtype (top panels), we found residual FM 1-43 staining in two clearly distinct cellular compartments, above and below the HC nuclei (visualized in bright field, central and merged right panels). In the apical compartments, we visualized FM 1-43 mostly clustered in vesicular and tubular structures. In the basal compartments, we found a more discreet but very distinct vesicular staining in vicinity to the basal cytoplasmic membrane, possibly coinciding with ribbon synapse location (magnified in bottom half panels). In contrast, mutant HCs (Figure 8B, lower panels) showed a much weaker vesicular/tubular apical staining with most clusters appearing much smaller than in wildtype NMs. Strikingly, in the basal compartments we found no residual staining (bottom half panels). To quantify the observed differences, we extensively imaged NMs (L1 to L5) in wildtype (*n* = 7) and mutant (*n* = 8) larvae at +21 hpt (Figure 8C). Using 3D reconstructions, we calculated the percentage of HCs with an apical and basal staining vs. total HCs (=% apico-basal clusters, bottom right graph). We found close to 4 times less apico-basal staining in mutant (open bar) vs. wildtype HCs (checkered bar, Wt = 83.65% vs. c*nr2^*upr/upr*1^* = 21.21% 9, *p* < 0.0001). Next, we considered apical staining separately which we further partitioned into small (diameter, *d* > 0.9 μm, top left graph) or large (*d* > 1 μm, top right graph) clusters of vesicles. Wildtype NMs (checkered bars) had ∼4 times more large apical clusters than *cnr2^*upr/urp*1^* (open bars) NMs (Wt = 4.171 vs. *cnr2^*upr*1^* = 1.282; *p* < 0.0001), but small clusters were predominant in mutant NMs (Wt = 21.29 vs. *cnr2* = 27.9; *p* = 0.001). The reduced residual apical staining and the increased number of small vesicles in mutant NMs suggested accelerated endosomal turn-over and/or increased apical endocytic activity. Furthermore, in the basal compartments of HCs, we seldomly found residual FM 1-43 in the mutant NMs (bottom left graph: Wt = 3.87 vs. cnr2 = 0.03, *p* = 0.0001), pointing to a possible alteration of synaptic endo- and exocytosis. Taken together, we observed that vesicular trafficking was severely altered within both apical and basal cell compartments of mutant HCs.

To confirm our findings, we established a time-course starting at +21 hpt (+21, +22, +23, and +24 hpt) imaging 4 dpf fixed larvae (*n* = 4/genotype/stage) that we had previously treated with a fixable analog of FM 1-43 (FM 1-43FX, Figures 8D–G). As previously observed, we found a strong reduction of the overall FM 1-43X staining in mutant NMs at +21 hpt (top views, 8D left panels). We quantified and averaged the clusters and found a strong decrease in the number of fluorescent clusters/NM (left graph) between wildtype (checkered bar) and mutant (open bar) NMs (Wt = 29 vs. *cnr2^*upr*1^* = 11.8; *p* = 0.0001). At +22 hpt (Figure 8E) when comparing to +21 hpt, number of clusters in wildtype was not significantly different, but dropped drastically in mutant NMs, increasing to a 7-fold difference between genotypes (Wt = 28.2 vs. *cnr2^ upr1^* = 3.9; *p* < 0.0001). From this stage onward, in mutant NMs we only found 3 to 4 residual FM 1- 43X containing clusters. In stark contrast, in wildtype NMs, we observed a slow and steady reduction of stained clusters/NM at +23 hpt (Figure 8E, Wt = 22.63 vs. *cnr2^*upr*1^* = 4.33; *p* < 0.0001), and +24 hpt (Figure 8G, Wt = 20.42 vs. *cnr2^ upr1^* = 3; *p* < 0.0001), suggesting that this approach was reliably reporting vesicular trafficking/recycling overtime. Taken together, the post-fixation imaging confirmed the strong reduction in residual FM 1-43 live staining that we had previously observed in animals lacking cnr2. Furthermore, the time course revealed a subtle and gradual reduction of FM 1-43X in wildtype NMs and highlighted a much more drastic drop in mutant NMs. Taken together, our FM 1-43 experiments demonstrated a strongly altered vesicular trafficking/recycling in HCs of animal lacking cnr2, possibly due to increased endo/exocytic activity in both the apical and basal compartments of HCs. Thus, we postulated that cnr2 function is intimately linked to vesicular trafficking in HCs, ultimately affecting proper LL and auditory function.

### Cnr2 Significantly Alters Swimming Behavior in Response to Sound Stimulation in a Light Dependent Manner

**FIGURE 9:**
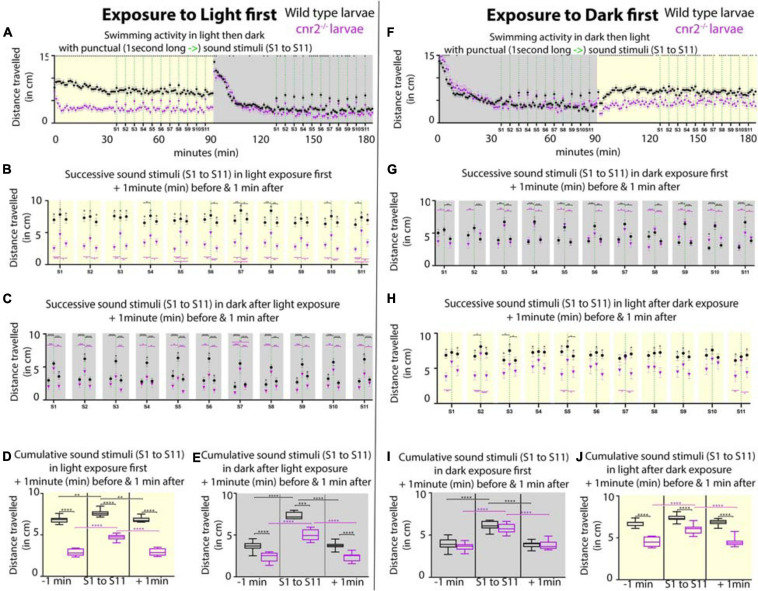
Swimming activity recorded in 6-day post-fertilization (dpf) wildtype and *cnr2*^*upr1/upr1*^ larvae after punctual sound stimuli (S1 to S11) during light or dark exposure. **(A)** Averaged swimming distances traveled/larva/min by wildtype (black dots) or mutant (magenta triangle) animals, that were first submitted to a 90-min light period (yellow box = maximum and constant intensity of 385Lux), followed by a 90-min dark period. At minute 36, larvae were exposed to a 1-s-long sound stimulus (S, vertical green dashed line at 450Hz) and then repeatedly at 5 min intervals (S1 to S11, yellow box). At S12, light was turned off and animals were left unstimulated for acclimation to dark for 35 min (gray box). At minute 126, sound stimulations were resumed (S1-S11, green dashed lines in gray box). **(B,C)** Averaged swimming responses in light (**B**, yellow box) and dark (**C**, gray box) periods during all successive sound stimulations (S1 to S11) showing the average distance traveled during the 1-min preceding, including, and following each individual sound stimulus (S1 to S11, green dashed lines). **(D,E)** Averaged swimming distances in light (**D**, yellow box) and dark (**E**, gray box) periods showing the compiled results for all 1-min preceding (-1 min), 1-min including (S1 to S11), and 1-min following (+1 min) the 11 sound stimulations. **(F–J)** Same modalities as in panel **(A–E)** except that animals are now first exposed to dark for a 90-min followed by a 90-min light period. Error bars represent the standard errors of the mean (SEM) and significance is indicated by * (* = *p* < 0.05, ** = *p* < 0.01*** *p* < 0.001, and **** *p* < 0.0001. Were omitted for clarity), comparing wildtype to mutant swimming activity, or as indicated by the color coordinated parentheses.

Next, we asked if auditory responses were affected in the absence of cnr2. To do so, we monitored swimming behaviors of wildtype and mutant animals that we submitted to repeated 1-s-long sound stimuli of constant amplitude (*S* = 450Hz) emitted at 5 min intervals (Figure 9, S1 to S11). To dissociate auditory from visual responses, animals were either first exposed to 90-min of constant light followed by 90-min of constant darkness (Figures 9A-E), or vice versa (Figures 9F-J). For animals first exposed to light, as we had previously demonstrated the baseline swimming activity (SA) for mutant animals was lower than for wildtype (Figure 9A, yellow box) (Acevedo-Canabal et al., 2019). In response to sound stimulation (green dashed lines in Figure 9A yellow box, Figures 9B,D) the SA was only slightly increased in wildtype by ∼14% (black, SA_*wt–*__1_ = 6.80 vs. SA_*wt–*__0_ = 7.65 cm/min, *p* < 0.0001), but by ∼63% in mutant larvae (magenta in D, SA_cnr__2__–__1_ = 2.87 vs. SA_cnr__2__–__0_ = 4.67cm/min, *p* < 0.0001). During the following dark periods upon sound stimulation (green dashed lines in Figure 9A gray box, Figures 9C,E), the increase in the respective SA were more comparable, ∼100% in wildtype (SA_*wt–*__1_ = 2.89 vs. SA_*wt–*__0_ = 5.79 cm/min, *p* > 0.0001) and ∼112% in mutant larvae (SA_cnr__2__–__1_ = 1.90 vs. SA_cnr__2__–__0_ = 4.03 cm/min, *p* < 0.0001). Taken together, this was suggesting a higher sensitivity of mutant larvae to sound, but mostly when also exposed to light. Interestingly, for animals first exposed to dark, the SA was not significantly different in wildtype and mutant larvae neither between, nor in response to sound stimulation (green dashed lines in Figure 9F gray box, Figures 9G,I). Thus, this corroborated that mutant larvae sensitivity to sound stimulation was higher when animals were also exposed to light. As expected during the following light periods (Figure 9F yellow box, Figures 9H,J), mutant traveled less than wildtype, but surprisingly more than mutant larvae that had not been previously exposed to sound (compare magenta in yellow boxes in Figure 9A vs. Figure 9F). This was true before, during, and after sound stimulation when comparing Figure 9D and Figure 9J (SA_cnr__2__–__1_ = 4.51 vs. SA_cnr__2__–__1_ = 2. *p* < 0. 0001, SA_cnr__2__–__0_ = 5.98 vs. SA_cnr__2__–__0_ = 4.67, *p* < 0. 0001, and SA_cnr__2__+__1_ = 4.52 vs. SA_cnr__2__+__1_ = 2.92 cm/min, *p* < 0.0001, respectively), but only for mutant larvae. In wildtype larvae with (black in Figure 9J), or without (black in Figure 9D) prior sound exposure, the SA was not different before, during, or after sound stimulation (SA_*wt–*__1_ = 6.80 vs. SA_*wt–*__1_ = 6.74 cm/min, SA_*wt–*__0_ = 7.65 vs. SA_*wt–*__0_ = 7.37 cm/min, and SA_*wt+*__1_ = 6.82 vs. SA_*wt+*__1_ = 6.87 cm/min, respectively). Thus, in light periods the SA of mutant larvae with prior exposure to sound stimulation was higher and closer to the wildtype SA, which was always unaffected by sound exposure. Taken together, animals lacking cnr2 appeared more sensitive to sound especially when also exposed to light, and less sensitive to light exposure after prior sound exposure. This was suggesting that both auditory and visual sensory systems were affected in the absence of cnr2.

Based on the perturbation that we observed in vesicle distribution at the ribbons in mutant retina, we hypothesized that mutant animals challenged by even slight variations in light intensity would not be able to fully readjust during gradual changes. To test this, we first reproduced our previously published experimental setup in which we recorded wildtype and mutant larvae SA after an initial 30 min adaptation to dark, followed by 4 successive 10-min light/10 min dark periods (Supplementary Figure 7A; Acevedo-Canabal et al., 2019). As expected, in mutant larvae (magenta squares) the SA was significantly reduced in light and increased in dark periods. Notably, the difference with wildtype SA (black dots) was not significant at the end of light and beginning of dark periods, suggesting that mutant larvae were slower to adapt to light, but also less challenged in dark. Next, instead of abrupt changes to full light or complete darkness, we progressively increased in 1-min increment to full light (100%) and then decreased to full darkness (0%) over the course of a 10 min period, for 4 successive cycles (Supplementary Figure 7B). Responses from one cycle to the next were highly consistent and showed that the swimming behavior was significantly different at all recorded time points between wildtype and mutant animals, for the exception of the minute of, and the minute immediately after full darkness. Mutant larvae swum significantly less at all other time points, implicating cnr2 function in detection of even slight light changes. Interestingly, the global trend in SA over all 4 cycles was identical in wildtype and mutant larvae, but at a much lower activity level in mutant larvae (compare black and magenta tracks/slopes in each cycle, SA_W__T_ ∼8 cm/min vs. SA_cnr__2_ ∼5 cm/min). This suggested that adaption to light changes was occurring in mutant larvae, but less effectively, thus corroborating findings in adult CB2-KOs mice (Cecyre et al., 2013). Taken together, this suggests a conserved role for cnr2 in adaptation to light that appears early during development and is maintained into adulthood.

## Discussion

### Cnr2 Expression in Physiologically Active and Mature HCs of the Inner Ear and the LL

We demonstrated strong *cnr2* transcriptional expression starting at 3 dpf in zebrafish larva in the mechanoreceptors or hair cells (HCs) of sensory epithelia (SE) of the inner ear and lateral line (LL). We further showed that the early development of those SE and the overall morphology of those organs was unaffected in *cnr2^*upr*1/upr1^* animals which are totally lacking cnr2 (Acevedo-Canabal et al., 2019). Thus, this was suggesting that cnr2 was not involved in early development of either organ, but rather had a role in maturation and/or physiology of HCs.

Little is known about the expression or function of either cannabinoid receptors (CNR1 or CNR2) in the ear and hearing. Audiograms and measurements of gap detection thresholds in Cnr1-KOs mice showed impaired hearing abilities at higher frequencies but enhanced gap detection thresholds (Toal et al., 2016). Those differences were attributed to Cnr1 function in the auditory brainstem where it is highly expressed (Kushmerick et al., 2004; Zhao et al., 2009; Zheng et al., 2015). For Cnr2, reports demonstrate the involvement in inflammation responses in the outer (Mimura et al., 2012) and middle ear (Oka et al., 2005). In the inner ear, a recent report documented spontaneous Cnr2 expression in the cochlear canal of adult rats (Martin-Saldana et al., 2016). Strong immunolabelling of Cnr2 was found in the *stria vascularis* (SV), and maybe more surprisingly in all inner HCs (IHCs), as well as in the afferent neurites and cell bodies of spiral neurons (Martin-Saldana et al., 2016). All these localizations were confirmed independently, and additional cochlear expression was found in the spiral ligament (SL), the outer HCs (OHCs) and a subset of support cells (SCs), namely the inner and outer pillar cells (IPCs and OPCs respectively) (Ghosh et al., 2018). Furthermore, this group showed that Cnr2 was co-localizing with markers of fibroblasts in the SL, basal cells in the SV, and ribbon synapse (Ribeye B or CtBP2) in the IHCs (Ghosh et al., 2018).

The underlying mode of action of Cnr2 in any of those cells/tissue remains to be determined. However, both groups demonstrated that Cnr2 expression was upregulated after Cisplatin exposure (Martin-Saldana et al., 2016; Ghosh et al., 2018). This was strengthening an earlier claim for an anti-apoptotic role for Cnr2 in cisplatin-treated cultured auditory cells (Jeong et al., 2007). In rats, intra tympanic pretreatments with Cnr2 agonist or antagonist (JWH015 and AM630 respectively) before cisplatin application, modulated cochlear inflammation, but pointed to a protective role of Cnr2 against several other ototoxic effects. First, it prevented apoptosis in OHCs. Second, it reduced loss of ribbon synapses in IHCs, and third, it maintained Na^+^/and K^+^-ATPase activity in SV and SL (Ghosh et al., 2018). All three aspects are strong contributors to optimal hearing. Furthermore, *in vivo* Cnr2- Knockdown (KD) showed that at least at lower frequencies, Cnr2 could mitigate cisplatin-induced hearing loss (Ghosh et al., 2018), thus strengthening the hypothesis of a protective role for Cnr2. Interestingly, presbycusis, or age-related hearing loss, is not only linked to HC loss, but also to SV degeneration with reduction of Na^+^ and K^+^-ATPase activity, the latter ultimately resulting in an energy starved cochlear amplificatory system (Schmiedt et al., 2002; Ding et al., 2018).

Similarly, a cardioprotective role for Cnr2 activation was described (Li et al., 2014), but it remains to be demonstrated if Cnr2 can offer protection against all forms of hearing losses. Furthermore, future therapeutic approaches will need to be Cnr2 specific and topical, because at least for Tinnitus (intermittent or constant phantom noise that has been mostly linked to auditory brainstem defects), cannabinoid treatments had negative results (Zheng et al., 2007, 2015; Smith and Zheng, 2016).

### Cnr2 Dependent Maturation of Ribbon Synapses

We found strong alteration in the sensory synapses of HCs of the LL in the pre- and post-synaptic elements, as well as in their alignment in 5 dpf *cnr2^*upr*1/upr1^* animals. Ribbon synapses formation and maturation have been well characterized in this very same context and at the developmental stages that we assessed (for review Kindt and Sheets, 2018). We found that all wildtype animals exhibited the previously described characteristic mature synapses in all HCs of NMs in the anterior LL (aLL), which develops first, as well as in the most rostral NMs of the posterior LL (pLL). As expected, we found more immature ribbon synapses in the most caudal wildtype NMs which are constantly added as the animal is growing (Thomas et al., 2015). In stark contrast in HCs of mutant animals, the NMs from the aLL and rostral pLL had mostly immature or even grossly misshapen ribbon synapses. Differences became less striking following an antero- posterior gradient in the pLL and were not always significant in the tail. Taken together, this was pointing to an involvement of cnr2 in the maturation of the sensory synapse.

Developmental maturation of ribbon synapses was previously extensively described in HCs (Michanski et al., 2019), but has never been linked to cnr2 function. The first mature and functional HCs can be found in the LL and inner ear as early as 3 dpf, even if the maturation process is ongoing in both structures into adulthood (Bever and Fekete, 2002; Whitfield et al., 2002). HC innervation also happens early and is extensively documented in the LL (Alexandre and Ghysen, 1999; Pujol-Marti et al., 2010; Pujol-Marti and Lopez-Schier, 2013), and it was demonstrated that innervation regulates ribbon synapses development and maturation (Suli et al., 2016). Interestingly, we did not find overt defects in the sensory or motor innervation of NMs, thus suggesting that the defects observed in the sensory synapses were HC autonomous, but this remains to be tested. However, we systematically found altered Maguk staining in the post-synaptic elements. Instead of being focalized in close vicinity to Ribeye b and aligned with it as we found and previously described in wildtype animals, it appeared diffuse and generally weaker in the mutant animals. Notably, we found similar alterations in the inner ear. The importance of physiological activity for maturation of the ribbon synapse is well known and has gained a lot of attention in recent years (for review Coate et al., 2019) especially in regard to synaptopathies (discussed below). Our work clearly pointed to a cnr2-mediated cross talk at the afferent synapse, thus offering a novel mechanistic link between maturation and activity.

### Cnr2 Modulation of Synapse Plasticity

We found a perturbed distribution of voltage- gated L- type Ca^+^ channels (Ca_v_1.3) at the active zone in *cnr2^*upr*1/upr1^* animals. The need for tight regulation of Ca^2+^ exchange at the sensory synapse had been extensively demonstrated in HCs of mammals (Platzer et al., 2000; Brandt et al., 2003; Brandt et al., 2005) but also Fish (Sidi et al., 2004; Sheets et al., 2012; Wong et al., 2014). An intimate relationship between ribbon size and Ca_v_1.3 channels density and distribution was repeatedly highlighted (Frank et al., 2010; Sheets et al., 2011, 2012). In physiological situations, bigger ribbons were shown to have more associated Ca_v_1.3 channels and larger calcium signals (Meyer et al., 2009; Ohn et al., 2016), but it was unclear if this ultimately translated into higher afferent activity. Additional work showed that when post-synaptic elements were unaffected and ribbon synapses’ size increased, clustering of Ca_v_1.3 channels was reduced at the active zone (Sheets et al., 2017), but both global and ribbon-localized calcium signals were increased, suggesting an alternate mode of Ca^2+^ levels regulation.

In conventional synapses, the endocannabinoid system (ECs) governs synaptic plasticity via a well described retrograde signaling that will result in short- or long-term depression of the presynaptic element (for review Castillo et al., 2012; Kendall and Yudowski, 2016). Short-term plasticity mostly involves direct G protein-dependent inhibition of Ca^2+^ influx through voltage-gated Ca^2+^ channels (VGCCs) (Kreitzer and Regehr, 2001; Wilson et al., 2001; Brown et al., 2003). CNR1 activation was shown to exert feedback inhibition of N, P/Q -type VGCCs in the CNS (Mackie et al., 1995; Twitchell et al., 1997; Guo and Ikeda, 2004), but also of L-type channels in smooth muscles (Gebremedhin et al., 1999) and bipolar neurons (Straiker et al., 1999). Furthermore, accumulating evidence demonstrated that CNR2 can also modulate synaptic activity in a variety of neurons, presumably through identical mechanisms (Xi et al., 2011; Stempel et al., 2016; Zhang et al., 2017). Thus, in HCs one possibility is that Cnr2 regulates mechanotransduction by modulating either global or local Ca^2+^ currents. This remains to be assessed and could be done by *in vivo* electrophysiology recording of HCs of the LL and inner ear using techniques which have been specifically developed in both organs in the developing and adult zebrafish (Olt et al., 2014, 2016a, 2016b). Measurements of whole cell and local Ca^2+^ currents in HCs of *cnr2^*upr*1/upr1^* animals could be further coupled to calcium imaging using some of the existing transgenic lines (Zhang et al., 2016). Finally, concomitant to Ca^2+^ influx, activation of K^+^ efflux is also directly controlled by ECs signaling (Mackie et al., 1995; Mackie, 2008). Interestingly, recent work highlighted how only a subset of HCs in each NM were active while others were silent due to an unknown regulatory mechanism linked to K^+^ levels (Zhang et al., 2018). Our discovery of a modulatory role of cnr2 at the HC ribbon synapse offers a tantalizing mechanism that begs testing.

ECs-dependent long-term synaptic plasticity involves inhibition of adenylyl cyclase and downregulation of the cAMP/PKA pathway which will ultimately result in inhibition of neurotransmitter release (Chevaleyre et al., 2007; Heifets and Castillo, 2009). We demonstrated that vesicles at the mutant ribbon synapse were uneven in size and shape, and that the tethering to the ribbon was irregular. The ribbon tethered vesicles represent the ready-to-release pool (RRP) available to support continuous transmission and constitute a timing system for delivering those vesicles to the plasma membrane in a synchronized manner (for review Matthews and Fuchs, 2010; Safieddine et al., 2012; Nicolson, 2015; Moser et al., 2020). Electron tomographic reconstructions of inhibited or stimulated HCs showed that depolarization was creating a gradient in size of the vesicles’ size (Lenzi et al., 2002), raising the possibility that the observed phenotype in the mutant HCs resulted from defective inhibition or regulation by Cnr2. This could be readily tested by capacitance measurements to establish if vesicular fusion is affected. HC synapse function can be further measured by electrophysiological recording from afferents which has been perfected in HCs of the LL (Trapani and Nicolson, 2010, 2011; Sheets et al., 2017).

We next showed that neurotransmitter vesicles in the vicinity of the ribbon synapse were affected in size, shape, and number at the active zone in cnr2 homozygotes. The mode of vesicle release is still a matter of debate in the field with evidence of both uniquantal release (UQR) and coordinated multiquantal release (MQR) in which coordinated synaptic vesicle (SV) exocytosis occurs at the ribbon active zone (AZ) (reviewed in Moser et al., 2020). However, what is agreed on is the necessity of appropriate coupling of exo- and endocytosis to allow the indefatigable synaptic transmission at ribbon synapses (Moser et al., 2020). Endocytosis is taking place in the peri-active zone which was demonstrated in the photoreceptor synapses (Wahl et al., 2013), and in the frog saccular HCs (Lenzi et al., 2002). Three endocytic mechanisms have been described (1) ultra-fast clathrin independent, (2) fast bulk endocytosis (Paillart et al., 2003), and (3) slow clathrin-mediated (Neef et al., 2014). Cnr2 could potentially modulate any or all those steps. Crossing the cnr2 mutants with loss-of-function mutant lines in proteins that have been involved in those mechanisms specific to the ribbon synapses like otoferlin will be helpful to address that (for review Safieddine et al., 2012).

Finally, we showed that the overall vesicular trafficking seemed accelerated in mutant HCs which unlike the wildtype larvae, had very little residual staining in the apical and basal compartments from 24 h post staining (hpt) onward. Notably, the HC specific apical endocytosis appeared unchanged, thus suggesting a difference in exo- endocytosis which could be restricted to the ribbon synapses but might also be due in part to constitutive membrane trafficking in other cell parts. To distinguish between those possibilities, a recently developed technique which is coupling FM 1-43 staining to photo-oxidation will allow to focus on synaptic trafficking only (Kamin et al., 2014).

A less well explored mode of action for ECs-was demonstrated for CNR1 which was found anchored in the external mitochondrial membrane, where it directed cellular respiration and energy production in murine neurons and participated in regulation of retrograde inhibition (Benard et al., 2012). Recent work in HCs of the LL highlighted how the ribbon size was modulated by mitochondria and how mitochondrial Ca^2+^ was participating in synaptic function (Wong et al., 2019). It remains to be demonstrated if Cnr2 is expressed in mitochondria in the vicinity of the ribbon synapse. If so, a provocative hypothesis to explore will be that mito-Cnr2 exerts a role like mito-Cnr1 in neurons, but specifically in sensory cells.

### Behavioral Changes in Cnr2 Mutant in Response to Sound Stimulation and Light Changes

When testing swimming behavior of animals lacking cnr2, we found that homozygote *cnr2^*upr*1/upr1^* larvae appeared more sensitive to sound when also exposed to light. Hyperacusis is a hyper-sensitivity to sound that was found associated with noise-induced damaged ribbon synapses in a mouse model (Hickox and Liberman, 2014). Synaptopathies get often undetected by audiograms because patients have normal hearing thresholds, but reduced supra-thresholds and it has been called the hidden hearing loss (Schaette and McAlpine, 2011). Additional unexpected finding in aged mice that had been young- exposed to noise at levels that produced only moderate threshold shift and no HCs loss, was that acute loss of synapses and peripheral terminal of the spiral neurons was presaging later ganglion cells losses (Kujawa and Liberman, 2015). This was suggesting that defective sensory synapses were setting the stage for neurodegeneration. This raises the possibility that we might be able to observe acute HCs degeneration in homozygote *cnr2^*upr*1/upr1^* adults. However, HCs regenerate in Fish unlike in mammals, and our earlier observation was that HC regeneration was unaffected in the larval LL. It will be of interest to verify if this remains true in the adult inner ear and LL.

Notably, mutant animals were responding differently to sound but only when in light. Pioneer work has recently shown a similar relationship between light and noise sensitivity in mice, demonstrating a higher sensitivity to noise in dark and showing the presence of a molecular clock in the cochlea, linking it to the circadian rhythm (Meltser et al., 2014). Unlike mice, zebrafish are diurnal which might explained the greater sensitivity to noise in light rather than in dark. The presence of a molecular clock in Fish HCs as well as its hypothetical modulation by cnr2 will be an interesting avenue to pursue. Interestingly, pinealocytes in the pineal gland, a major player in the control of circadian rhythms have ribbon synapses that presented an altered morphology in mutant larvae (LCC and MB unpublished), which warrants further exploration.

We had previously shown that *cnr2^*upr*1/upr1^* larvae were behaving differently in light and dark (Acevedo-Canabal et al., 2019) which we reproduced here, and we further showed that mutant larvae were slower to adapt to light changes. Anecdotal reports of improved night vision after cannabis ingestion (West, 1991; Russo et al., 2004) and glare recovery impairment experiments (Adams et al., 1978) have mostly linked altered vision to CNR1. However, the retina of adult mice showed no alteration neither in Cnr1-KOs nor Cnr2-KOs, and only the latter required more adaptation time to light (Cecyre et al., 2013). Cnr1 protein expression in adult retina was extensively described in several cell types and in various species (for review Bouchard et al., 2016) including goldfish (Cottone et al., 2013). In the developing retina, expression of Cnr1 along with other components of the endocannabinoid signaling pathway (Zabouri et al., 2011b; Cecyre et al., 2014a) were described in embryonic (Buckley et al., 1998) and postnatal rat (Zabouri et al., 2011a), as well as embryonic chick (Leonelli et al., 2005). Pharmacological manipulations with Cnr1 and Cnr2 agonists and inverse agonists suggested an involvement of ECs in the retinothalamic development which needs further investigation (Duff et al., 2013). However, the expression of Cnr2 in the adult retina remains controversial because of debated specificity of available Abs (Baek et al., 2013; Cecyre et al., 2014b) and has not been explored in the developing retina, which needs to be addressed.

We found subtle but consistent alterations in the retina of mutant *cnr2^*upr*1/upr1^* larvae. Most ribbons had incomplete or poorly defined arciform densities where the base of the ribbon is anchored. The main component of this electron dense ultra-structure is Bassoon, which when functionally disrupted results in free floating ribbons (Dick et al., 2003). The active zone had a blurry and weakly delineated appearance and the surrounding vesicles appeared scattered, interspersed, and of variable size. Taken together, this was suggesting perturbation of the vesicular trafficking in the presynaptic active zone that needs further investigation. Ribbons in photoreceptors are highly dynamic and their size is well known to vary with illumination (Schmitz, 2009) and diurnal signals in mice and fish (Vollrath and Spiwoks-Becker, 1996; Adly et al., 1999; Balkema et al., 2001; Spiwoks-Becker et al., 2004; Hull et al., 2006; Lagnado and Schmitz, 2015). Behavioral experiments allowing to discriminate between the Cnr2 exerted regulation of individual sensory systems (pineal gland, retina, inner ear, and LL) will be highly informative. The deciphering of each relative contribution will be paramount in providing a holistic understanding of the Cnr2 role(s) in the regulation of individual and combined sensory inputs.

### Cnr2 Activation: A Potential Therapeutic Route for Synaptopathies

Auditory or cochlear synaptopathies are a specific type of sensory hearing loss in which HCs appear intact and can detect sound stimulation, but are unable to transmit the signal at the sensory synapse (Moser et al., 2013). The origin can be genetic like loss-of function mutations in the *Vesicular glutamate transporter-3 gene (VGLUT3)* causing progressive non-syndromic hearing loss (Ruel et al., 2008), that was independently identified in zebrafish (Obholzer et al., 2008) and in mice (Seal et al., 2008). Zebrafish HCs are remarkably similar to mammalian HCs (Coffin et al., 2004) and there is a strong gene conservation from Fish to mammals (Vona et al., 2020). Thus, a growing number of mutant lines are providing excellent tools for hearing disorder modeling (Whitfield et al., 2002; Nicolson, 2005). More recently, it became evident that damage to ribbon synapses represent a highly prevalent form of acquired sensory hearing loss (Wan and Corfas, 2015; Eggermont, 2017), mainly caused by aging (Sergeyenko et al., 2013) and noise-induced damage (Kujawa and Liberman, 2015; Liberman and Kujawa, 2017). Aging as well as overexposure to noise, both result in dramatic swelling of the afferent dendrites at the ribbon synapse which can be prevented by pretreatment with AMPA/Kainate antagonists of the post-synaptic glutamate receptors as well as by pharmacological blockage of glutamate release, suggesting glutamate excitotoxicity (Ruel et al., 2007). Notably, sensitivity to aminoglycosides might also be related to damage in ribbon synapses as a primary effect which will eventually be followed by HCs death depending on the dose (Ruan et al., 2014). Furthermore, and as discussed above, cisplatin ototoxicity was affecting ribbons synapses and was mitigated by Cnr2 pharmacological manipulations (Ghosh et al., 2018), thus offering a promising therapeutic approach to correct or prevent synaptopathies.

## Conclusion

We showed for the first time a clear relationship between the maturation and function of the ribbon synapse (RS) and the endocannabinoid system (ECs) in two sensory systems of a developing vertebrate. Our work explored how a loss-of-function mutation in the *cnr2* gene was linked to defective swimming responses triggered by sound and light in zebrafish mutant larvae. First, we demonstrated for the first time the expression of *cnr2* in HCs of the LL and the sensory patches of the inner ear in larval zebrafish, which was concordant with previous expression studies in adult rodents and derived auditory cell lines (Jeong et al., 2007; Martin-Saldana et al., 2016; Ghosh et al., 2018). Second, we showed strong perturbations in several components of the sensory synapse in the mutant *cnr2^*upr*1/upr1^* larvae which became increasingly obvious as HCs were maturing. We noted similar perturbations in the RS in the mutant retina. Third, we linked these morphologic alterations of the RS to an altered cellular physiology by showing that the vesicular trafficking in HCs was strongly perturbed. Fourth, we illustrated differences in swimming activity in response to sound or light stimulation in mutant *cnr2^*upr*1/upr1^* larvae, therefore, underlining the relevance of the observed phenotypic differences. Taken together, we presented alterations linked to the absence of cnr2 in developing zebrafish larva at the ultra-structural, structural, cellular, and physiological levels and ultimately linked them to behavior. Our work strongly suggested a pivotal role for CNR2 in the regulation of mechanotransduction in HCs. Furthermore, our data implied that the CNR2 mediated regulation might be common to other sensory systems.

## Materials and Methods

See Table 1.

**TABLE 1:** Key resources table.

Reagent type, species or resource	Designation	Source/reference	Identifier/dilution
Genetic reagent (Danio rerio)	Wildtype		TAB-5 (Tubingen x AB)
Genetic reagent (Danio rerio)	Wildtype		NHGRI-1
Genetic reagent (Danio rerio)	cnr2-KO	Acevedo-Canabal et al., 2019	*cnr2upr1/upr1*
Gene (*Danio rerio*)	*cnr2*	Ensembl	ENSDARG00000039970.7
Commercial assay or kit	RNA Clean & Concentrator	Zymo Research	Cat#R1016
Commercial assay or kit	DNA Clean & Concentrator	Zymo Research	Cat#RD4014
Commercial assay or kit	SuperScript III One-Step RT PCR Kit	Sigma Aldrich	Cat#12574-026
Chemical compound	FM 1-43	Invitrogen	Cat#T35336
Chemical compound	FM 1-43X	Invitrogen	Cat#F35355
Antibody	Mouse Monoclonal anti Ribeye b	Gift from Teresa Nicolson Lab	1/2000
Antibody	Rabbit Polyclonal anti Ribeye a	Gift from Teresa Nicolson Lab	1/5000
Antibody	Rabbit Polyclonal anti Ribeye b	Gift from Teresa Nicolson Lab	1/5000
Antibody	Rabbit Polyclonal anti-Ca_v_1.3a	Gift from Teresa Nicolson Lab	1/1000
Antibody	Anti-K28/86	NeuroMab, Davis	1/500
Antibody	Znp1	Hybrodoma Bank	1/200
Antibody	Zn12	Hybrodoma Bank	1/50
Antibody	Alexa-Fluor 488 anti-Rabbit	Thermo Fisher	Cat # A11034 d = 1/1000
Antibody	Alexa-Fluor 568 Anti-mouse	Thermo Fisher	Cat #A11031 d = 1/1000
Machine/Equipment	Zebrabox^®^	Viewpoint, France	
Software, algorithm	GraphPad Prism	GraphPad Software	www.graphpad.com
Software, algorithm	Zen Lite 2.6	Zeiss	www.zeiss.com
Software, algorithm	Zen Blue	Zeiss	www.zeiss.com
Software, algorithm	FIJI	<PMID>PMID:22743772</PMID>	
Software, algorithm	ZebraLab	Viewpoint, France	
Microscope	Zeiss Axio Imager Z2 Coupled to Confocal Laser Scanning Microscope (LSM800)	Zeiss	
Microscope	Inverted Epifluorescence Axiovert	Zeiss	
			

### Ethics Statement

We carried-out experiments in accordance with the guidelines and protocols approved by the IACUC (#A880110) of the University of Puerto Rico – Medical Sciences Campus (UPR-MSC).

### Zebrafish Care and Husbandry

We performed animal care and husbandry following previously published protocols (Westerfield, 1993) and NIH guidelines. We used zebrafish (*Danio rerio*) for all experiments, which we raised and maintained in the UPR-MSC Satellite Fish Room facility according to standard procedures as recommended (Westerfield, 1993). We raised and kept all fish at 28°C on 14:10 h light/dark cycles on a recirculating system (Techniplast^®^). Water supplied to the system was filtered by reverse osmosis (Siemens) and maintained at neutral pH (7.0–7.5) and stable conductivity (1,000 μS/cm) by adding sea salt (Instant Ocean^®^). This water is referred to as system water (SW). After each cross, we collected, rinsed, and raised embryos for the first 24-h post fertilization (hpf) in SW with methylene blue (0.2%). After 24-hpf, we raised fertilized and anatomically normal embryos in SW at 28°C on 14:10-h light/dark cycles until 6-day post fertilization (dpf). We only used larvae devoid of anatomical abnormalities and exhibiting upright swimming for further experiments. We did all developmental staging according to (Kimmel et al., 1995).

### Zebrafish Lines

We bred, maintained, and staged wildtype (TAB-5 = Tubingen × AB, and NHGRI-1) and *cnr2^*upr*1/upr1^* mutants as previously described (Acevedo-Canabal et al., 2019). To genotype fish, we fin-clipped them once they reached adulthood (=3 months old) and digested fins in 30 μL 50mM NaOH (Sigma Aldrich) at 95°C for 20 mins (min). Next, we added 30 μL 100 mM Tris-HCl, and PCR-amplified fragments from the *cnr2* target region using the following primers (Forward: 5′-GACCACACAAGAGCAGAAAGC-3′, and Reverse: 5′-GACGATCCAACCAGGTTTTG-3′) as stated previously (Acevedo-Canabal et al., 2019).

### Whole-Mount *in situ* Hybridization (WISH)

We extracted total RNAs using trizol (TRI-Reagent, Sigma Aldrich) from 5dpf larvae to synthetize sense and antisense probes. We used the SuperScript III One-Step retro-transcription kit (Sigma 12574-026) and designed gene specific primers (GSPs) for *cnr2* (Forward: GATCAAGAAGCTACGACTGTGC, and Reverse: ACTACCACTCACTGCCGGAT) with T7 (atgctaatacgactcactatagggaga) and T3 (atgcattaaccctcactaaaggga) promoter sequences attached to the forward and reverse primers, respectively. The expected amplicon length was 1,080bp. We performed WISH as described previously with minor modifications (Thisse and Thisse, 2014). First, we dechorionated embryos and rehydrated in methanol/10X PBS gradient solutions (75% methanol, 50% methanol, and 25% methanol) for 5 mins (min) and then rinsed in PBST (1%Tween 20) for 5 min. We bleached animals older than 24 hpf with 30% H_2_O_2_ (Sigma) for approximately 20 min. Next, we digested larvae older than 2 dpf with 2 mg/ml of Proteinase K (Ambion) for 7 min, and later fixed at room temperature (RT) (4% paraformaldehyde) for 45 min. After rinsing 5 × 5 min in Blocking Buffer for WISH (1X P880110BS, 0.1% Tween 20, 0.1% BSA, 1% DMSO), we pre-hybridized larvae in hybridization mix (HM) (50% Formamide, 5X SSC, 1 mg/mL Yeast RNA, 50 μm/mL Heparin, 0.1% Tween 20, 5mM EDTA, 9mM Citric Acid in DEPC treated water) for 4-6 h. Next, we hybridized all specimens with the pre-heated antisense probe (final concentration = 1 ng/μl) overnight (O/N) at 65°C. We washed larvae in a series of HM solutions in 2X SSC (75%, 50%, 25%) for 10min each, followed by two 30 min washes of 0.2X SSC, all at 65°C. Next, we rinsed all animals in 0.2X SSC gradient solutions in PBST (75%, 50%, and 25%) for 10min each at RT. We pre-incubated all specimens in Blocking Buffer for 4-6 h and then incubated in pre-absorbed anti-DIG-AB against fish powder in blocking buffer (1/3000) O/N. Finally, we rinsed all animals in PBST 6 × 15 min followed by two 5 min washes in Alkaline Phosphatase Buffer (APB) (100mM Tris pH9.5, 50 mM MgCl2, 100 mM NaCl, 0.1% Tween 20, levamisole). Revelation was performed using BM Purple (100%) in the dark.

### Immunohistochemistry (IHC)

We fixed animals O/N (PFA 4%) and washed 3 × 5 min in PBST (PBS 1X, 0.1% Tween 20). Next, we stored at −20°C in methanol (100%). When ready to perform IHC, we rehydrated specimens using serial dilutions (MeOH 75%/PBST 25%, MeOH 50%/PBST 50%, MeOH 25%/PBST 75%, PBST 100%) for 10min each. We then replaced PBST with cold Acetone for 7 min at −20°C, and then rehydrated in PBST with 3 × 5 min washes. We digested larvae with 1 mg/mL of collagenase in Blocking Buffer (PBST, 10% Goat Serum, 0.1% BSA) for 35min. Next, we washed all specimens with Blocking Buffer 5 × 5 min, after which we pre-incubated them in fresh Blocking Buffer for 4-6 h followed by primary antibody incubation O/N. Next, we rinsed larvae in PBST 4 × 15 min and pre-incubated them in Blocking Buffer for 4-6 h before incubating O/N in secondary antibody. Next, we rinsed larvae 4 × 15 min in PBST and mounted them for imaging in poly-Aquamount (PolySciences).

The following affinity-purified primary antibodies were a kind gift from Dr. Teresa Nicolson (Sheets et al., 2012) and generated against *Danio rerio*: mouse monoclonal against Ribeye b (amino acids 12-33; Open Biosystems, Huntsville, AL, United States) and rabbit polyclonal against Ribeye a (amino acids 1-466; Proteintech, Chicago, IL, United States), Ribeye b (amino acids 4-483; Proteintech), and Ca_v_1.3a (amino acids 42-56; Open Biosystems). We used the K28/86 (NeuroMab, Davis, CA, United States) to label MAGUKs. Secondary antibodies used were Alexa-Fluor 488 Anti-Rabbit and Alexa Fluor 568 Anti-Mouse.

### FM 1-43 and FM 1-43X Hair Cell (HC) Staining and Live Imaging

We used FM 1-43 live dye (Invitrogen, #T35356) and its fixable analog (FM 1-32FX, Invitrogen, #F35355). For either, we exposed larvae for 30 s (sec) at the desired stage to 3 μM FM 1-43(X), rinsed 3 × 30 s in SW. For the fixable version and at the desired time point post-treatment we anesthetized in ice cold water and subsequently fixed animals (PFA 4%, O/N). Next day we washed larvae 3 × 5 min in SW before mounting them on slides (see above in ICH).

For live imaging, we mounted larvae at the desired post treatment time point using 2% low-melting agarose (LMA) in bottom coverslip chambers. Imaging was performed on an inverted Axiovert (Zeiss) creating z-stacks (in 1 μm steps) from an identified NM in the pLL (L5) using the 63X DIC oil-immersion objective. The first stack was recorded as soon as animals were mounted in LMA, and each following stack was recorded in 30min intervals for a period of 24 h. We recorded the first 4-h immediately post FM 1-43 staining. We used these z-stacks and time-point recordings to re-construct a 24-h post-treatment timeframe.

### Semi-Thin and Ultra-Thin Sections for Transmission Electron Microscopy (TEM)

Larvae were prepared as described previously (Behra et al., 2009). Briefly, larvae were fixed overnight in 2.5% glutaraldehyde (Sigma) and 4% paraformaldehyde prepared from paraformaldehyde (Sigma) in 0.1M sodium cacodylate buffer (Sigma). Larvae were then rinsed and post-fixed 1h at room temperature in reduced osmium (1:1 mixture of 2% aqueous potassium ferrocyanide) as described previously (Kujawa and Liberman, 2015). After post-fixation, the cells were dehydrated in ethanol and processed for Epon (Sigma) embedding. Semi-thin sections (300 nm) were cut and collected on a glass slide, and subsequently stained using toluidine blue (Sigma). The analysis and imaging were done on an inverted Zeiss Axiovert200M. Ultra-thin sections (80 nm) were cut on a Reichter-E ultramicrotome, collected on copper grids and stained with lead citrate (Sigma) for 2 min. Sections were then examined with a CM 10 Philips electron microscope at 80 kV. We performed serial sectioning to follow the same ribbon in three to five sections on the same grid.

### Confocal Microscopy

For confocal imaging we processed larvae as previously described (Behra et al., 2009). Briefly, IHC processed larvae were mounted in poly-Aquamount (Polysciences) on slides prepared with fenestrated tape in 3 layers to avoid squashing them. Acquisitions were performed on a Zeiss Axio-Imager Z2 coupled to a confocal laser scanning microscope (LSM800) with a Pan-Apochromat 63 × /1.40 oil-immersion objective. It is to be noted that staining in the mutants were often considerably weaker and when keeping the same setting used for wildtype NMs and ears, we would often have no signal at all making the comparison impossible. Therefore, we adjusted the laser setting as necessary to obtain an optimal image for each NM. When comparing we would integrate the acquisition differences in our final assessment.

### Image Acquisition and Post-processing

For FM 1-43 staining, we acquired images on an inverted Axiovert (Zeiss) with Axiovision and post-processed as needed with the Zen Lite 2.6 software. Confocal images were acquired as described above with maximal projections of z-stacks created and analyzed using Zen Blue software. Final figures were created with Adobe Photoshop and Illustrator.

### Swimming Activity Tracking

To measure swimming activity, we conducted behavioral assays using a previously described setup (Colon-Cruz et al., 2018). In brief, 24 h prior to the experiment, we plated one individual larva per well in a 48-well plate (Greiner bio one, CELLSTAR^®^) with 450 μl of SW. The next day and prior to the experiment we topped up each well to 500 μl of SW and performed a health check. Only healthy (with inflated swim bladder, no obvious external damage and swimming upright) were used for the behavioral testing. We placed the 48-well plate into the Zebrabox^®^ (Viewpoint, France), which is an isolated recording device with a top camera and infrared light-emitting base where we controlled temperature, light intensity, and sound/vibration stimulations. We recorded swimming activity 1 min per minute using the Viewpoint tracking software (ZebraLab).

### The Photo-Dependent Response (PDR) and the Gradual-PDR (g-PDR)

In the photo-dependent response (PDR) as developed in Colon-Cruz et al. (2018), we first habituated larvae for 30 mins (adaptation/incubation) in the dark (I_min_ = 0 Lux = 0%) followed by a sharpen transition to light at maximum intensity (I_max_ = 385 Lux = 100%) for 10 min followed by 10 min of dark. We proceeded with four successive cycles (80 mins) for a total recorded time of 110 mins.

In the gradual-PDR (g-PDR), we modified the luminosity intensity gradient minute per minute to reach the maximum (I_max_) or minimum (I_min_) light intensity at the end of each 10-min interval ramping from 0 to 100% in light and back to dark from 100 to 0%. Specifically, we changed the light intensity by a factor of ±10% (=38.5 Lux) per minute. For both PDR and s-PDR assays, we performed four independent experiments (*n* = 83 wildtype and 94 mutant larvae).

### The Acoustic Evoked Response (AR) in Light or Dark

For the acoustic evoked response (AR), experiments were either started with larvae exposed to light for 90 mins followed by a dark period of 90 mins, or to dark first followed by light for the same time periods. Starting at minute 36, larvae were submitted to a recurring 1-s sound stimulus of average intensity (=450 Hz) every 5 mins for a total of 12 stimulations (S1-S12). At stimulation S12 (=minute 90), they were also switched to the alternate light/dark state and then left unstimulated for the following 35 mins. Starting at minute 126, sounds stimulations were resumed every 5 min. For each assay (light or dark first), we performed three independent experiments (L/D: *n* = 68 wildtype and 62 mutant larvae; D/L: *n* = 51 wildtype and 41 mutant larvae).

### Statistical Analyses

We analyzed averaged total traveled distances per larva (with a minimum of triplicate experiments and 24 animals/treatment) in GraphPad Prism (v.8). All results were binned into 1-min intervals and error bars represent the mean standard error of the mean (SEM). Statistical differences between direct comparisons were calculated using multiple t-tests controlling the effect of the correlation among the number of fixed repeated measures. We performed two-way analysis of variance (ANOVA) in graphs when two or more groups were compared simultaneously. Differences with *p* < 0.05 were considered significant (^∗^).

## Data Availability Statement

The raw data supporting the conclusions of this article will be made available by the authors, without undue reservation.

## Ethics Statement

We carried-out experiments in accordance with the guidelines and protocols approved by the IACUC (#A880110) of the University of Puerto Rico – Medical Sciences Campus (UPR-MSC).

## Author Contributions

MB, LC-C, and RR-M conceived the experiments and wrote the manuscript. LC-C, RR-M, AS-C, JC-V, AT-T, and S-JL performed the experiments. LC-C, RR-M, and AS-C did confocal acquisitions and participated in realizing the figures of the manuscript. RK and MB performed all EM imaging. GY, BM, SB, OR, and GV provided intellectual input for technical approaches and maturation of the manuscript. OR, GV, and MB provided intellectual and technical expertise in support of all experiments.

## Conflict of Interest

The authors declare that the research was conducted in the absence of any commercial or financial relationships that could be construed as a potential conflict of interest.

## Supplementary Material

Supplementary Figure 1 Lateral views of whole larvae after *in situ* hybridization. **(A)** Whole 3 dpf larvae hybridized with a *cnr2* sense probe. **(B)** Whole 3 dpf larva hybridized with a cnr2 antisense probe. **(C)** Whole 5 dpf larva hybridized with a *cnr2* antisense probe. Scale bar = 100 microns.

Supplementary Figure 2 YOPRO-1 staining of HCs in live animals during development and regeneration. **(A)** Wildtype (top panels) and *cnr2*^*upr1/upr1*^ mutants (bottom panels) showing one cranial (O, left) and two trunk NMs (L1, middle and right). **(B)** Average number of HC/NM at each respective developmental stage (2 to 7 dpf) in wildtype (dark gray) and *cnr2*^*upr1/upr1*^ (light gray) larvae. **(C)** Average number of regenerated HCs/NM in wildtype (dark gray) and *cnr2*^*upr1/upr1*^ (light gray) larvae after synchronous ablation with copper treatment (=0-h post-treatment, hpt) and subsequent counts performed at +24, +48, and +72-hpt.

Supplementary Figure 3 Motor and sensory innervation in 5 dpf wildtype and *cnr2*^*upr1/upr1*^ larvae. **(A)** Top view of cranial NM (IO2) from the anterior lateral line (aLL) in wildtype (top lane) and mutant (bottom lane) larvae that were immunolabelled with Znp1 (magenta) to stain motor innervation, a HC-specific AB against myosin 7 (Myo7), and counterstained with DAPI. **(B)** Top view of cranial NM (O) in larvae that were immunolabelled with Zn12 (magenta) to stain sensory innervation, an AB against Ribeye b (green) in presynaptic ribbon synapses, and counterstained with DAPI. Scale bars = 20 microns.

Supplementary Figure 4 Transmission electron microscopy (TEM) images of cone pedicles showing ribbon synapses in triads in the retina of 5dpf wildtype and *cnr2*^*upr1/upr1*^mutant larvae. **(A,D)** In wildtype retina, most ribbon synapses have clearly defined arciform densities (blue arrows) in close vicinity to the presynaptic plasma membrane densities (pm, blue arrowheads). They are surrounded by numerous vesicles of ∼similar size (white arrowheads). **(B,C,E,F)** In *cnr2*^*upr1/upr1*^ retina, most ribbons synapses have poorly defined or incomplete arciform densities (magenta arrows) and presynaptic plasma membrane (magenta arrowheads). The surrounding vesicles appear scarcer and more uneven size (white arrowheads). Scale bar in panel **(A)** representative for all images: = 50 nm.

Supplementary Figure 5 Live imaging of wildtype and *cnr2*^*upr1/upr1*^ NMs 1-h post FM 1-43 treatment (hpt). Top views of NM in the pLL (L5) in wildtype (top panels) and *cnr2*^*upr1/upr1*^(bottom panels), showing FM 1-43 (magenta in left and right columns) that penetrated a subset of HCs and dispersed throughout the cell, and brightfield view of the NMs (DIC, center and right columns). Scale bar: = 20 microns.

Supplementary Figure 6 FM 1-43 treatment time course in live wildtype and *cnr2*^*upr1/upr1*^ NMs from +30 mins to +8 hpt. Top views of NM in the pLL (L5) in wildtype (top panels) and *cnr2*^*upr1/upr1*^(bottom panels), showing FM 1-43 (magenta in left and right columns) that penetrated a subset of HCs and dispersed throughout the cell, and brightfield view of the NMs (DIC, center and right columns). Scale bar: = 20 microns.

Supplementary Figure 7 Recording of individual swimming behaviors of 6-day post-fertilization (dpf) wildtype and *cnr2*^*upr1/upr1*^ larvae. **(A)** Averaged distance traveled per minute by 6 dpf wild-type (black circles) and *cnr2*^*upr1/upr1*^ (magenta squares) larvae submitted to four successive cycles of 10 min of alternating light periods (white boxes) and dark periods (gray boxes) after a 30-min habituation period to dark. **(B)** Averaged distance traveled per minute by larvae submitted to 4 successive cycles of 10min gradual light intensity increase (0-100%) followed by gradual light intensity decrease (100-0%) after a 30-min habituation period to dark. Error bars represent the standard errors of the mean (SEM) and statistical significance is indicated by^∗^ (^∗^*p* < 0.05 and ns is omitted for clarity).

Supplementary Movie 1 Zn12-Rib-Wt.

Supplementary Movie 2 Zn12-Rib-cnr2.

Supplementary Movie 3 Rib-Maguk-macula Wt.

Supplementary Movie 4 Rib-Maguk-macula-cnr2.
