# Supplementary figures and images for: Cnr2 Is Important for Ribbon Synapse Maturation and Function in Hair Cells and Photoreceptors
Source: Front Mol Neurosci. 2021 Apr 20;14:624265. doi: 10.3389/fnmol.2021.624265 (PMC8093779; doi:10.3389/fnmol.2021.624265)

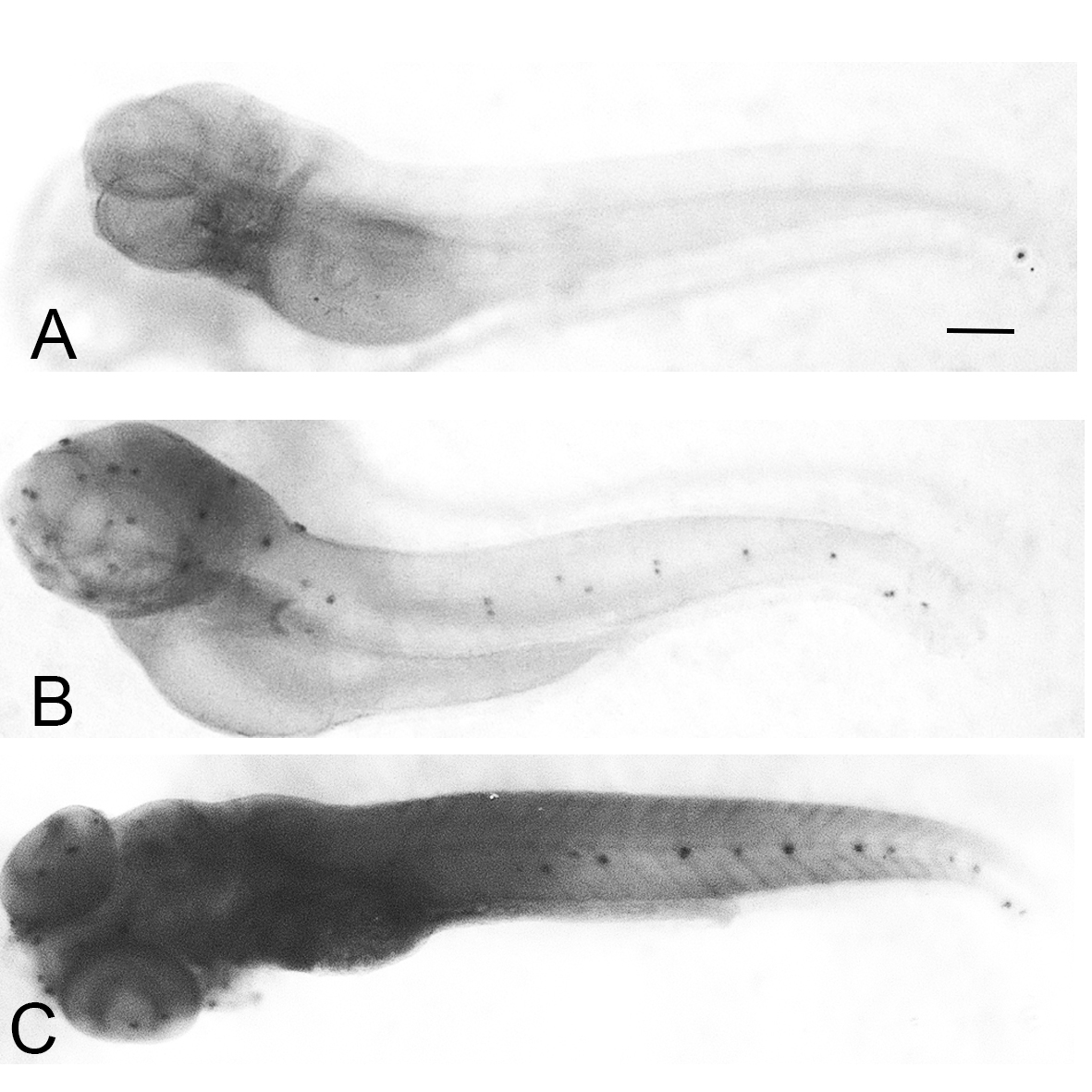

Supplement: Supplementary Figure 1 — Lateral views of whole larvae after in situ hybridization. (A) Whole 3 dpf larvae hybridized with a cnr2 sense probe. (B) Whole 3 dpf larva hybridized with a cnr2 antisense probe. (C) Whole 5 dpf larva hybridized with a cnr2 antisense probe. Scale bar = 100 microns. [file Image_1.jpg]

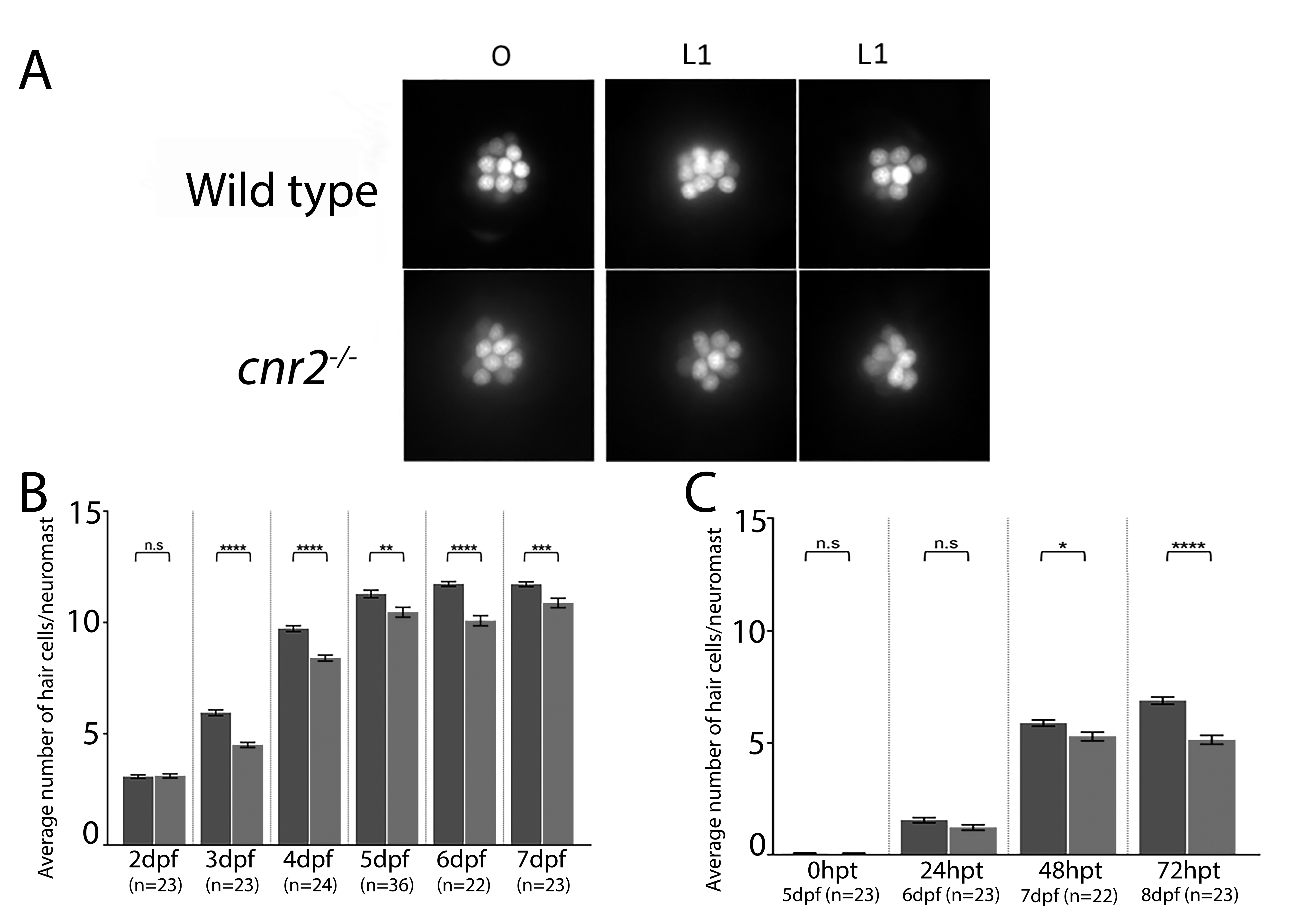

Supplement: Supplementary Figure 2 — YOPRO-1 staining of HCs in live animals during development and regeneration. (A) Wildtype (top panels) and cnr2upr1/upr1 mutants (bottom panels) showing one cranial (O, left) and two trunk NMs (L1, middle and right). (B) Average number of HC/NM at each respective developmental stage (2 to 7 dpf) in wildtype (dark gray) and cnr2upr1/upr1 (light gray) larvae. (C) Average number of regenerated HCs/NM in wildtype (dark gray) and cnr2upr1/upr1 (light gray) larvae after synchronous ablation with copper treatment (=0-h post-treatment, hpt) and subsequent counts performed at +24, +48, and +72-hpt. [file Image_2.jpg]

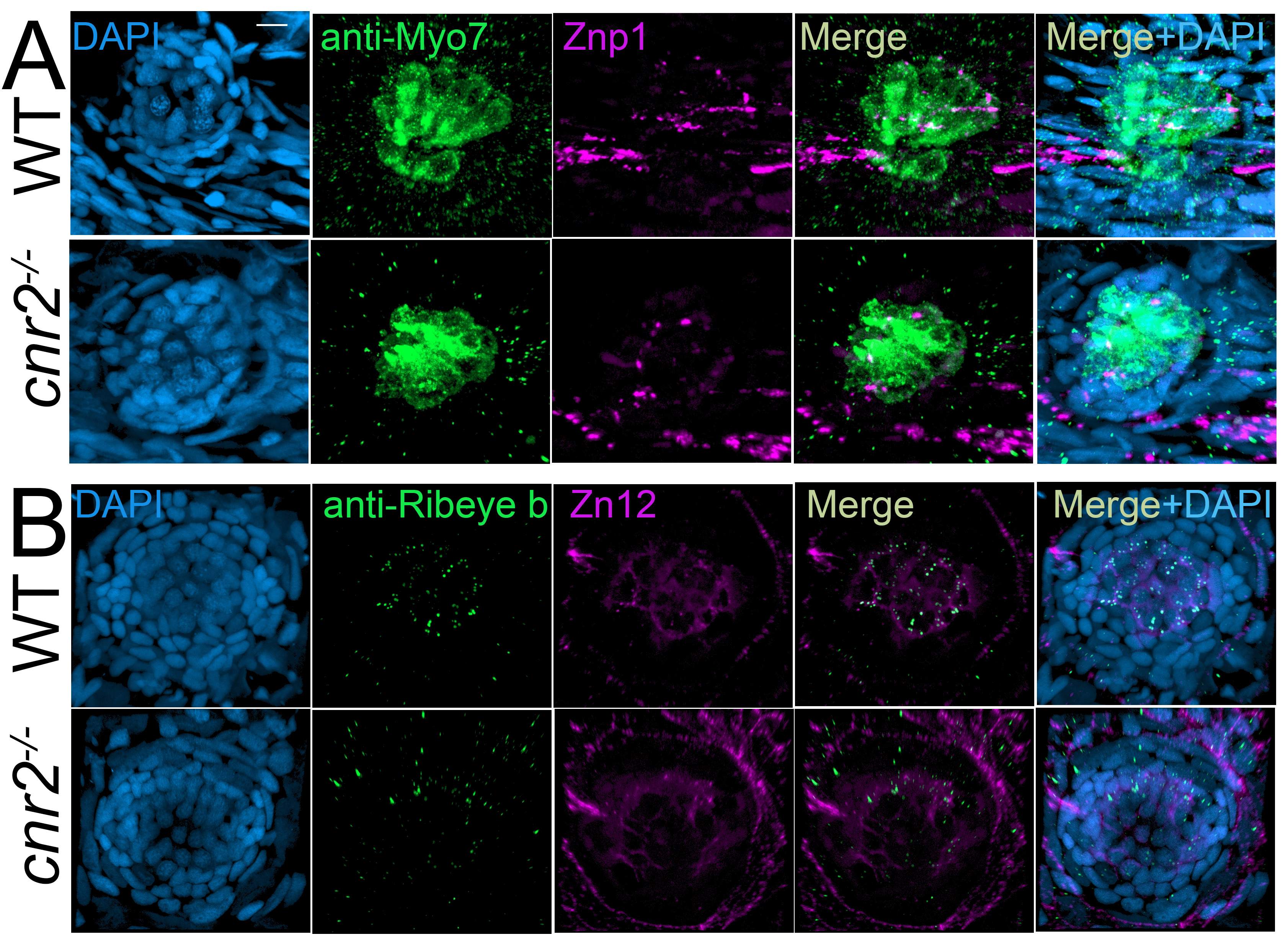

Supplement: Supplementary Figure 3 — Motor and sensory innervation in 5 dpf wildtype and cnr2upr1/upr1 larvae. (A) Top view of cranial NM (IO2) from the anterior lateral line (aLL) in wildtype (top lane) and mutant (bottom lane) larvae that were immunolabelled with Znp1 (magenta) to stain motor innervation, a HC-specific AB against myosin 7 (Myo7), and counterstained with DAPI. (B) Top view of cranial NM (O) in larvae that were immunolabelled with Zn12 (magenta) to stain sensory innervation, an AB against Ribeye b (green) in presynaptic ribbon synapses, and counterstained with DAPI. Scale bars = 20 microns. [file Image_3.jpg]

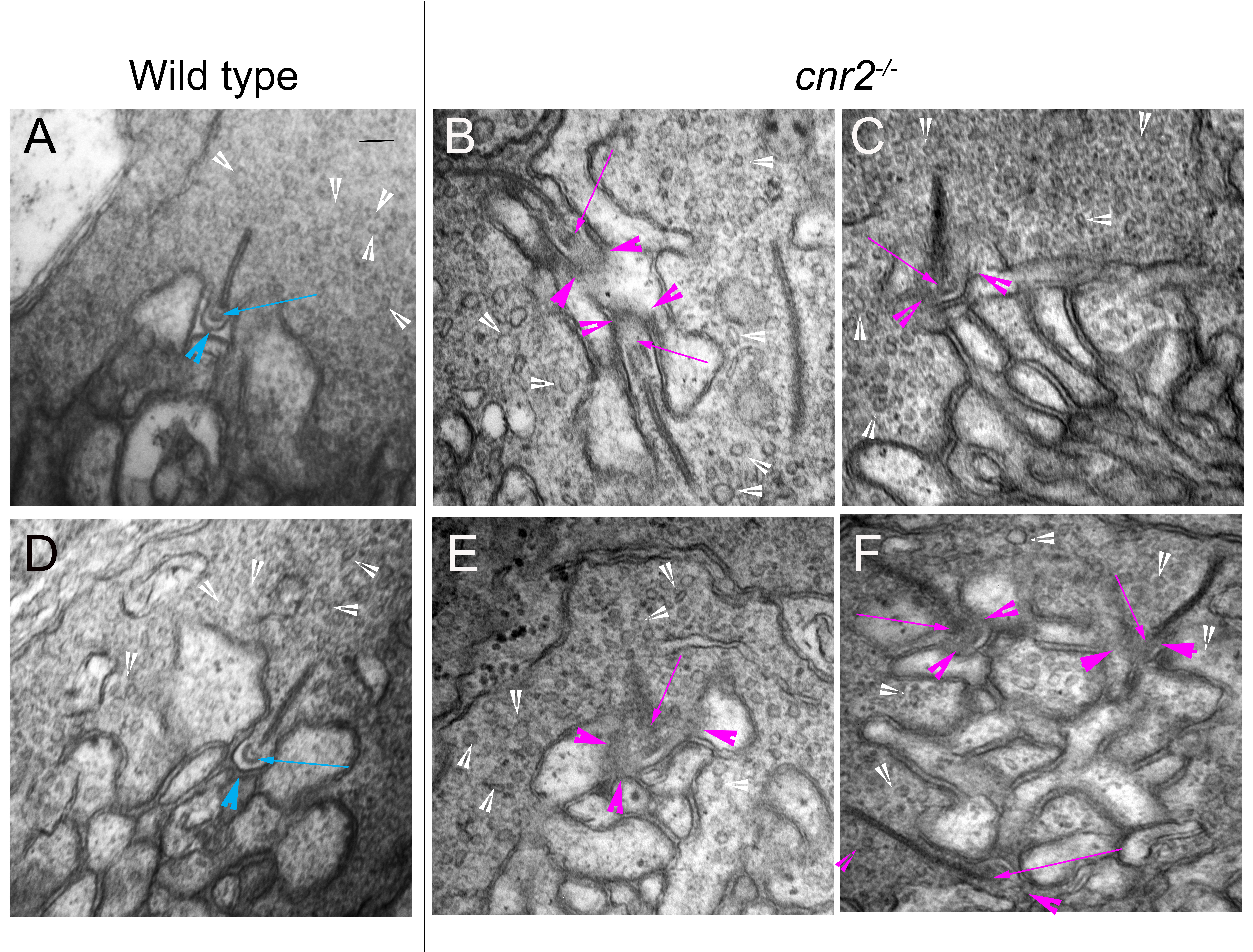

Supplement: Supplementary Figure 4 — Transmission electron microscopy (TEM) images of cone pedicles showing ribbon synapses in triads in the retina of 5dpf wildtype and cnr2upr1/upr1mutant larvae. (A,D) In wildtype retina, most ribbon synapses have clearly defined arciform densities (blue arrows) in close vicinity to the presynaptic plasma membrane densities (pm, blue arrowheads). They are surrounded by numerous vesicles of ∼similar size (white arrowheads). (B,C,E,F) In cnr2upr1/upr1 retina, most ribbons synapses have poorly defined or incomplete arciform densities (magenta arrows) and presynaptic plasma membrane (magenta arrowheads). The surrounding vesicles appear scarcer and more uneven size (white arrowheads). Scale bar in panel (A) representative for all images: = 50 nm. [file Image_4.jpg]

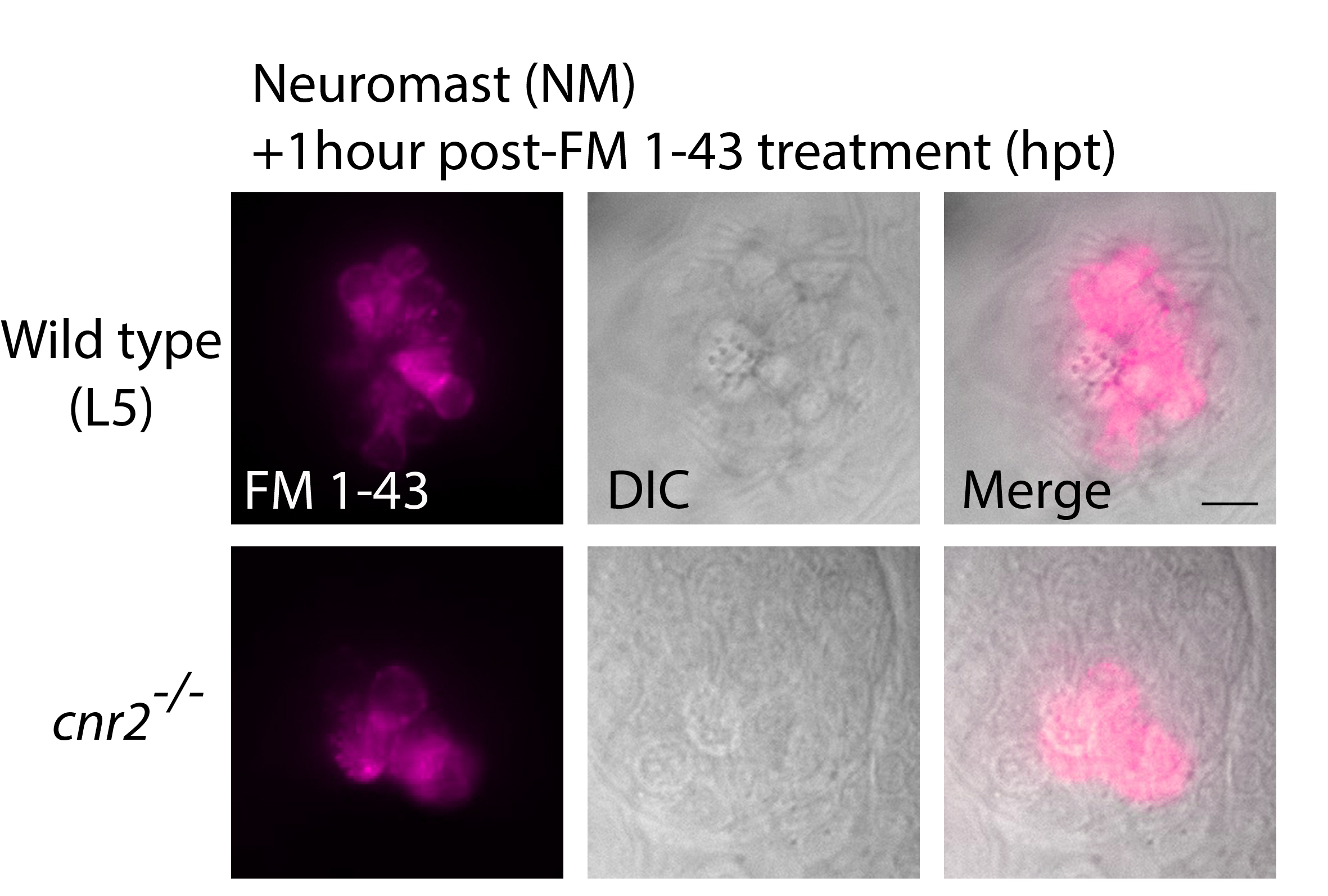

Supplement: Supplementary Figure 5 — Live imaging of wildtype and cnr2upr1/upr1 NMs 1-h post FM 1-43 treatment (hpt). Top views of NM in the pLL (L5) in wildtype (top panels) and cnr2upr1/upr1(bottom panels), showing FM 1-43 (magenta in left and right columns) that penetrated a subset of HCs and dispersed throughout the cell, and brightfield view of the NMs (DIC, center and right columns). Scale bar: = 20 microns. [file Image_5.jpg]

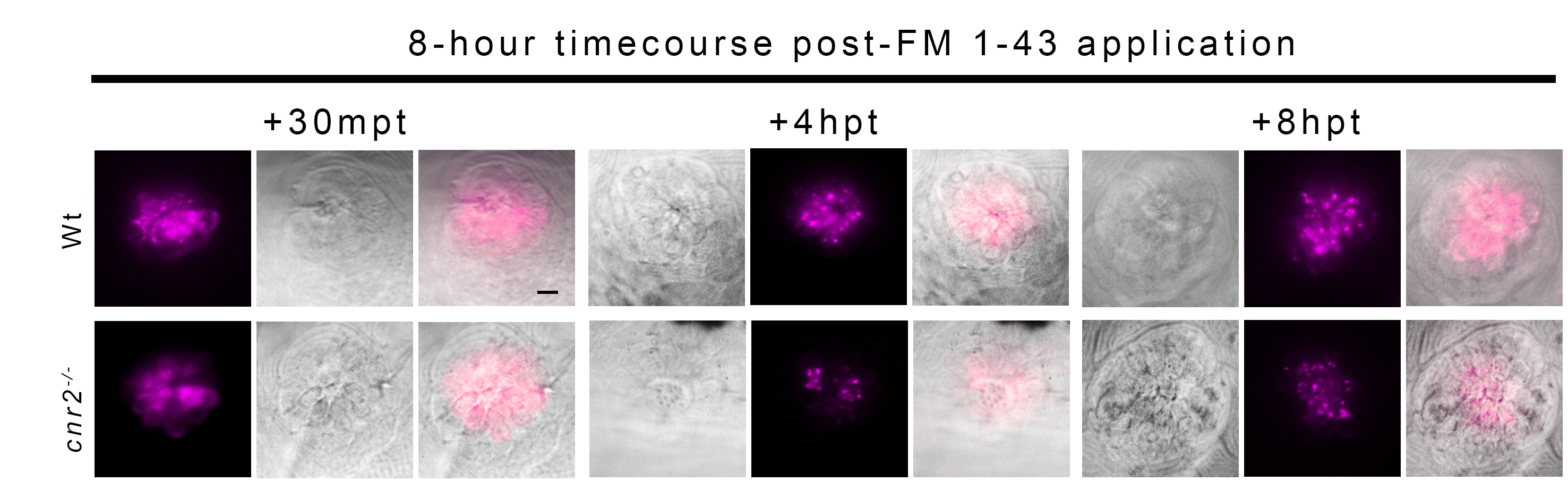

Supplement: Supplementary Figure 6 — FM 1-43 treatment time course in live wildtype and cnr2upr1/upr1 NMs from +30 mins to +8 hpt. Top views of NM in the pLL (L5) in wildtype (top panels) and cnr2upr1/upr1(bottom panels), showing FM 1-43 (magenta in left and right columns) that penetrated a subset of HCs and dispersed throughout the cell, and brightfield view of the NMs (DIC, center and right columns). Scale bar: = 20 microns. [file Image_6.jpg]

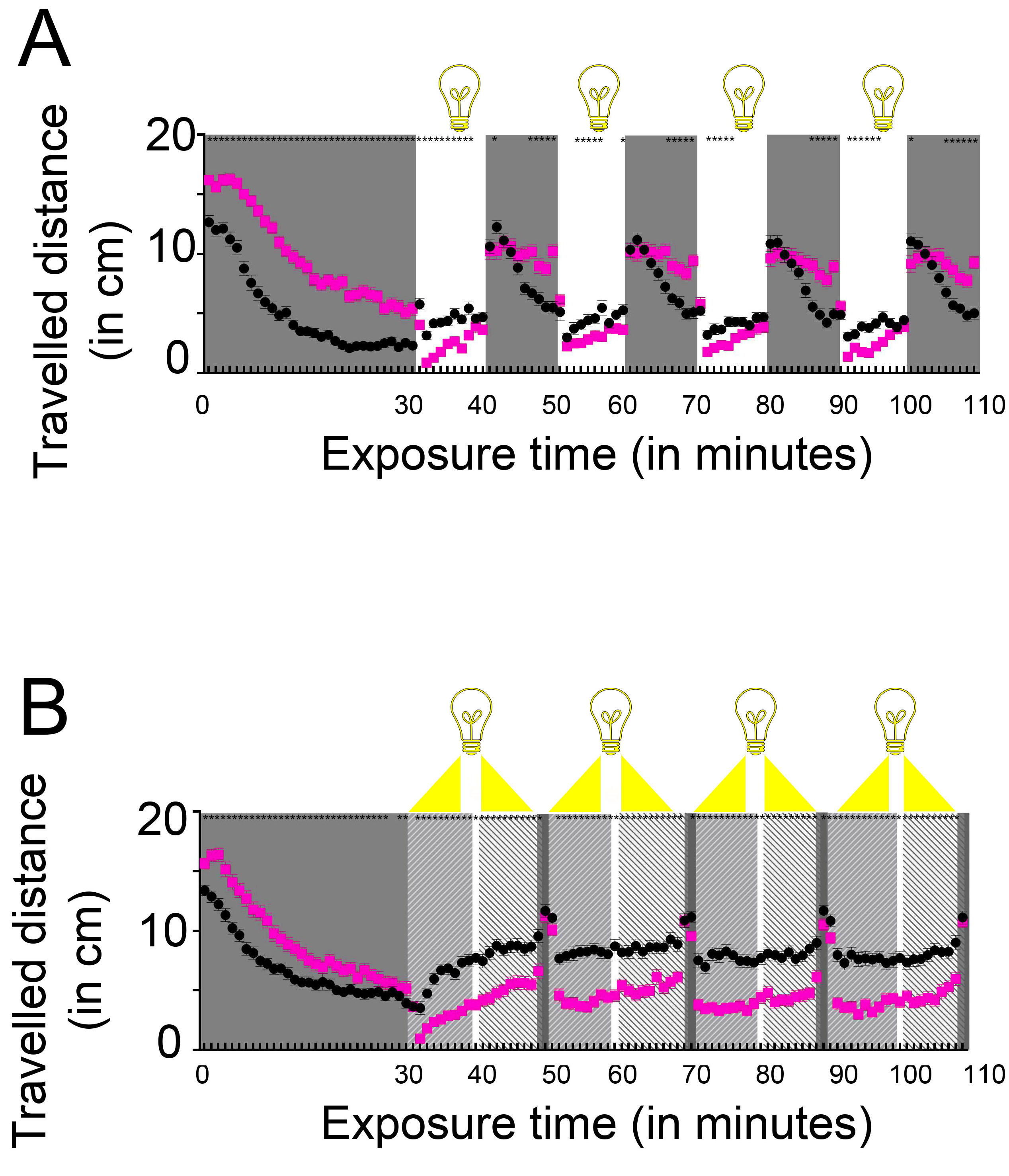

Supplement: Supplementary Figure 7 — Recording of individual swimming behaviors of 6-day post-fertilization (dpf) wildtype and cnr2upr1/upr1 larvae. (A) Averaged distance traveled per minute by 6 dpf wild-type (black circles) and cnr2upr1/upr1 (magenta squares) larvae submitted to four successive cycles of 10 min of alternating light periods (white boxes) and dark periods (gray boxes) after a 30-min habituation period to dark. (B) Averaged distance traveled per minute by larvae submitted to 4 successive cycles of 10min gradual light intensity increase (0-100%) followed by gradual light intensity decrease (100-0%) after a 30-min habituation period to dark. Error bars represent the standard errors of the mean (SEM) and statistical significance is indicated by∗ (∗p < 0.05 and ns is omitted for clarity). [file Image_7.jpg]
